# Environmental heterogeneity plays **a** bigger role **than** diet quality **in** driving divergent California sea lion population trends

**DOI:** 10.1371/journal.pone.0324108

**Published:** 2025-11-05

**Authors:** Ana Lucía Pozas-Franco, David A. S. Rosen, Andrew W. Trites, Francisco J. García-Rodríguez, Claudia J. Hernández-Camacho

**Affiliations:** 1 Marine Mammal Research Unit, Institute for the Oceans and Fisheries, University of British Columbia, Vancouver, British Columbia, Canada; 2 Instituto Politécnico Nacional, Centro Interdisciplinario de Ciencias Marinas, La Paz, Baja California Sur, México; National Cheng Kung University, TAIWAN

## Abstract

While the global population of California sea lions (*Zalophus californianus*) is increasing, regional trends show a decline in the Gulf of California (GoC, Mexico) and an increase in the Channel Islands (CI, U.S.) over the last 40 years. The drivers of these divergent trends remain unclear, but previous pinniped studies suggest that differences in diet quality—rather than prey abundance—may play a role. We therefore examined how California sea lion population trajectories relate to diet quality, specifically looking at diet energy density and diet diversity. Using population and diet data from 1980 to 2020 for sea lions in the GoC and CI, we found no simple relationships between population trajectories and diet quality over time at either the local or regional level. Energy densities of sea lion diets were similar between the two regions, but GoC sea lions consumed a more diverse range of prey (n = 88 vs. 23 main prey taxa) dominated by benthic species and schooling fishes, while CI diets consisted mainly of schooling fishes and squid. We also found that GoC sea lions ate more benthic prey and less schooling fish during the 2014–2016 heatwave—decreasing their overall diet energy density. This shift coincided with a temporary population decline in the CI but had variable effects on GoC populations. Overall, our findings suggest that regional population trends are influenced by complex ecological factors beyond diet quality alone, highlighting the need to consider environmental variability and prey composition when assessing the resilience of sea lion populations to climate-driven changes.

## Introduction

California sea lions (*Zalophus californianus*) are widely distributed along the Pacific coast of North America, from British Columbia, Canada to the Gulf of California, Mexico, but only reproduce on certain islands (rookeries) along the southern coast of California (U.S.), the Mexican Pacific, and the Gulf of California [1,2]. Most of the global population (80%) breeds in California where numbers increased at an annual rate of 2.9% between 1964–2014 [3]. The remainder of the population (20%) breeds in Mexico, where—with a few exceptions—most rookeries show a declining trend. In the Gulf of California, sea lion populations experienced on average a 2% decline per year between 1984–2015 [4,5].

Along the southern Pacific coast of the U.S., California sea lions breed almost exclusively at four rookeries that form part of the Channel Islands, which vary in size from 3,000–60,000 individuals [6]. This population has grown steadily since the 1980s, but has experienced short-term population declines in some years associated with increased sea surface temperatures as seen during the 2012–2016 marine heatwave [6]. The population has since recovered, totaling 111,713 sea lions in 2019 [6]. In contrast, breeding sea lion populations in the Gulf of California, Mexico, are distributed among 13 rookeries of varying size from 400–6,000 individuals [1,2]. Although these rookeries vary in population growth, most show a declining trajectory since the 1980s. Only one rookery in the southernmost Gulf of California – Los Islotes – is considered to have a population that has been increasing since 1979 [7].

The underlying factors causing a divergence in California sea lion population trajectories in the Channel Islands compared to the Gulf of California are still unknown [5]. Possible contributing factors that are generally known to affect other marine mammal population levels include regional differences in prey availability, pollution (both chemical and noise), disease, biotoxins, fishing gear entanglements, anthropogenic mortality (disturbance, legal and illegal shooting), and migration [8–11]. Of these contributing factors, regional differences in diets associated with environmental conditions have been identified as the most likely contributor to population trajectories in California sea lions and other marine mammal species [4,12–15].

Previous studies have generally focused on the negative effects that short-term reductions in prey *abundance* have on California sea lion numbers at rookeries in the Channel Islands [3,16,17] and the Gulf of California [7,14,18–21]. Sharp declines in quantities of primary prey species available to sea lions are known to occur during El Niño events in California when warm water causes prey to remain at inaccessible depths, leading to increased pup mortality [13]. However, El Niño events do not appear to have a comparable direct effect on the Gulf of California sea lion populations where the response to warming events depends on location, population size, and regional dynamics [5]. This leads to the question of whether changes in the *quality* of prey (rather than changes in *quantity*) might better explain differences that have occurred in sea lion numbers over a longer timeframe [22], as suggested for Steller sea lions [23–27].

Diet quality can be assessed in many ways. Two main metrics of diet quality are diet energy density (an important aspect of the nutritional value of prey species) and diet diversity (i.e., the variety of species that compose the diet). These diet characteristics can affect the nutritional status of individuals, their reproduction and survival rates, as well as their susceptibility to disease and predation [25,26]. Based on broad trends observed among different marine mammals, a diet dominated by a few energy-rich species would be hypothesized to support a growing population, while a switch to a more diverse diet of energy-poor species would be expected to cause population declines [12,15,24]. It has also been demonstrated that changes in environmental conditions can alter the diet composition and ‘quality’ of prey species available to predators [25,26]. However, it is not known how such differences or changes in diet quality may influence the population dynamics of California sea lions in the Gulf of California compared to the Channel Islands.

To investigate the effect of diet on population trajectories, we used long-term data from rookeries in the Channel Islands and the Gulf of California spanning 1980–2020. We quantified diet quality using average energy density and two measures of diet diversity, and tested for relationships between these metrics and sea lion population trends over time. We also assessed diet changes in the Gulf of California before and after a major warming event. These comparisons contribute to a better understanding of how environmental and dietary factors shape California sea lion population dynamics, helping inform conservation and management policies across their range in Mexico and the U.S.

## Materials and methods

### Population and diet data

**Fig 1:**
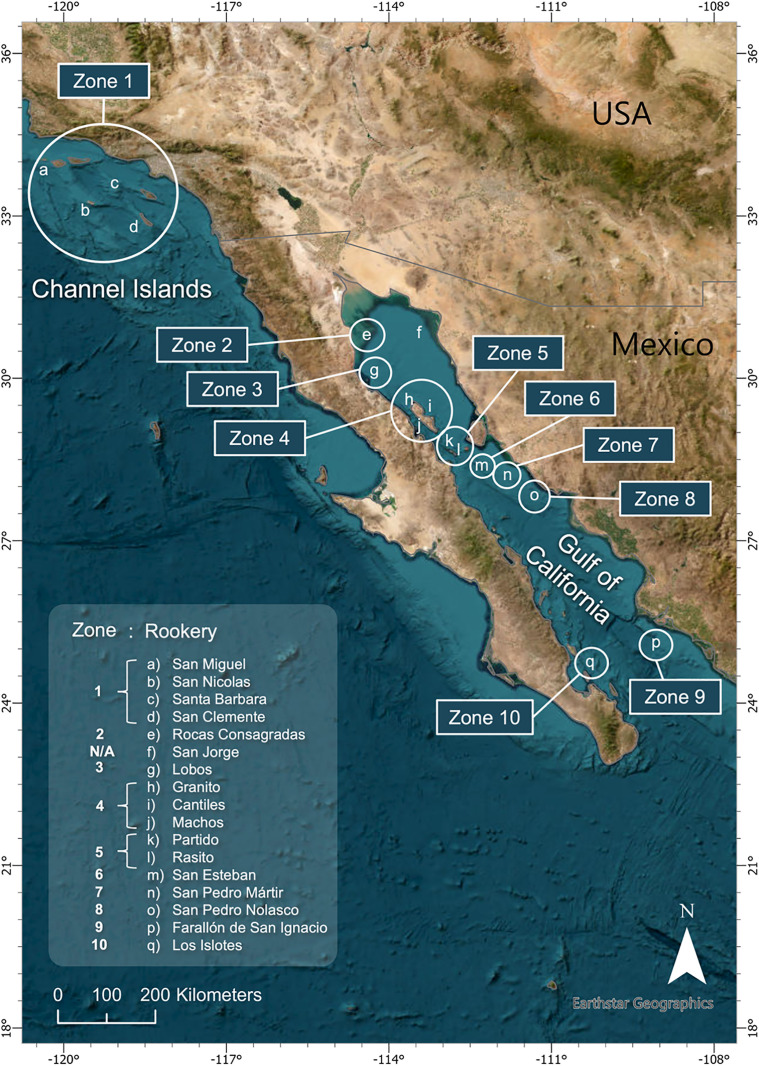
Map of the California sea lion rookeries and designated Zones for this study. Study sites included the four rookeries in the Channel Islands (a–d: San Miguel, San Nicolas, Santa Barbara, San Clemente) designated as Zone 1, and the 13 rookeries along the Gulf of California **(e–q)**, and their respective Zones (2–10). Circles indicate rookeries within the indicated Zone. Rookery f (San Jorge) did not have diet data available and was omitted from further analysis. For reference, the distance between the northernmost rookery of Roca Consagradas (Zone 2), and the southernmost rookery of Los Islotes (Zone 10) is 823 km.

Our meta-analysis focused on the four California sea lion rookeries in the Channel Islands (San Miguel, San Clemente, Santa Barbara, and San Nicolas), and 12 of the 13 rookeries along the Gulf of California (Fig 1). We omitted the San Jorge rookery due to a lack of available data and also had insufficient data to include the west coast of the Baja California Peninsula.

We quantified sea lion *diet quality* using estimates of average energy density and two measures of diet diversity: Shannon’s Index and number of main prey species. We compared the measures of diet quality within and between geographic regions (Channel Islands and Gulf of California) and tested for relationships between rates of population change and diet quality over time.

Available population and diet data for California sea lion rookeries in the Channel Islands and the Gulf of California from 1980–2020 were gathered from published and unpublished sources (S1 and S2 Tables in S1 File). All data (population counts and diet data) used in this study were collected during the sea lion breeding season from May to August because, 1) most of the data available was from this season, 2) population data from this season captures the maximum number of sea lions present that year at a rookery by including newborn pups, and 3) we wanted to minimize the potential confounding effects that might be introduced by seasonal changes in diet when comparing diets across years and areas. However, in the few cases when breeding season data were unavailable for a particular rookery and year, we relied on data from another season if available.

### Diet data

Diet data refers to data on the occurrence of identified prey from hard parts in sea lion scat samples collected at rookeries. In most cases, prey were identified to the species level in the original sources, although in some cases, identification was limited to higher taxonomic levels (e.g., squids, rockfish). For simplicity, we refer to all taxonomic groupings as “prey taxa”. Available data was used to assess diet quality, which we characterized using measures of diet diversity and diet energy density (detailed below). Both diet characteristics were calculated using data originally reported as frequency of occurrence of prey taxa (FO) [28], or as an index of importance (IIMP) of prey taxa (Gulf of California data only) [20,29].

To test the relationship between diet quality and population change, we ideally needed matching diet data for the years with available population data. However, diet data were sparse, unevenly distributed over time, and only available for certain rookeries and years. Additionally, in some cases data were reported as a single mean spanning several years (e.g., Santa Barbara Island, 1981–1995; S2 Table) in S1 File. To address these limitations and make use of all available data, various data processing methods were employed prior to conducting analyses.

### Processing diet data

Only prey taxa with FO values ≥5% were included in this dataset because: 1) this was the cut-off available from most of the original data sources, and 2) it served to highlight the main prey items. We also applied a ≥ 5% cut-off to IIMP values to maintain consistency across the data (this is the same IIMP cut-off previously used by others [30]. Note that while some literature used a cut-off of IIMP values ≥10% [20], we were able to apply a ≥ 5% IIMP value cut-off because we had access to the raw diet data (provided by co-author García-Rodríguez). ‘Non-identified’ species reported in the IIMP data were deemed not useful for this analysis and were therefore excluded. After collecting raw data from published and unpublished sources, all diet results in this study refer to main prey taxa from FO or IIMP of ≥5%.

To be able to compare diet data between rookeries and years, remaining FO and IIMP values were standardized to sum to 1.0 (or 100%) within each year of data by dividing each reported value by the total FO or IIMP for that rookery and year. This yielded *modified* frequency of occurrence (MFO; [31]) and *modified* importance index (MIIMP) values which we subsequently used to calculate diet diversity and energy density.

### Calculating diet diversity

Two measures of diet diversity were used: 1) the total number of main prey taxa recorded in the diet, and 2) diet diversity calculated using the Shannon Index [32] from either MFO or MIIMP values for individual species (where a higher resulting H-index indicates greater species diversity). The Shannon Index was selected because it accounts for both species’ richness and evenness, allowing less common prey taxa to contribute to diversity estimates and reducing bias toward dominant taxa. Values of average diet diversity using the Shannon Index were calculated for each rookery and year when data was available and used in subsequent analyses (S3 Table in S1 File).

### Calculating (weighted) diet energy density

The energy density of each prey species (or prey taxa) was recorded in kilojoules per gram of wet weight (kJ/gww), using data from a pelagic species traits database [33]. If an energy density value was not available at the species level, an average energy density value from the species’ family (or in a few cases a closely related family) was used instead to approximate energy density. We then calculated the *weighted* average diet energy density (to account for relative appearance of each prey taxa in the diet) by multiplying the MFO or MIIMP (expressions of proportion in the diet) by the respective energy density of that prey taxa (in kJ/gww). Summing these values gave an average weighted diet energy density value for each rookery and year, which was used in subsequent analyses (S3 Table in S1 File).

### Population data

The population counts we used represent the maximum number of individuals observed during the sea lion breeding season (July–August) and include all age and sex classes. These counts were intended to capture peak seasonal abundance, when both pups and adult females are present at rookeries. All reported sea lion counts from 1980–2018 from the Gulf of California rookeries were obtained from [14]. Counts were made from boat surveys and included numbers for each age and sex class with a correction factor of 28% to account for females that were missed or at sea during the count [34]. Population counts for the Channel Islands were available from 1980–2019 and were sourced from [3] for 1980–2014, from [35] for 2015, and from [6] for 2016–2019 (S1 Table in S1 File). These counts had already been corrected for pups that were obscured from vision and for adult females that were foraging during the census. In cases where multiple sources reported population counts for California sea lions in the Channel Islands for the same rookery/year, the higher estimate was used whenever the discrepancy likely resulted from a method presumed more accurate—such as aerial photographic surveys versus boat-based counts [36].

### Calculating population change

To test the relationship between population and diet quality, we calculated rates of population change for subsets of years with available diet data at each rookery. Using regression analysis, we estimated population changes immediately associated with single or grouped years with diet data (i.e., year-rookery grouping) by incorporating population counts from a set number of years before and after the diet data. Since the range of years incorporated into each rookery-year diet data point varied, a set of rules were established to define the number of years before and after the diet data span that were included in the calculation of population change, depending on how many years of diet data were included for that grouping. Further details on calculating population change are provided in the S1 Methods. In general, population trends were estimated from data that spanned the period of diet data by at least an additional year on either side.

### Grouping data

#### Creating Zones and Zone-era groupings.

To investigate the relationship between population changes and diet quality, we had to ensure the independence of the data points on both a temporal (both sequential and non-sequential data) and geographic scale (closely related rookeries). This involved grouping rookery diet data (energy density and diet diversity averages) and population data (population change averages over a set of years) together into non-continuous sets of years into rookery-year groupings (more details in S1 Methods). We then created sets of matching population and diet data sets averaged across related geographic areas (Zones) and non-continuous time periods (eras): Zone-era groupings.

As previously noted, neither population nor diet data were continuous across years, and the timing of the data was not consistent across rookeries, resulting in the previously described rookery-year groupings. Values for the eventual ‘Zone-era’ data groupings were created by averaging the population change of each rookery-year grouping, and the respective diet quality values (energy density and diversity) within a geographic Zone (described below) for each set of number of years or ‘era’ with available data.

**Fig 2:**
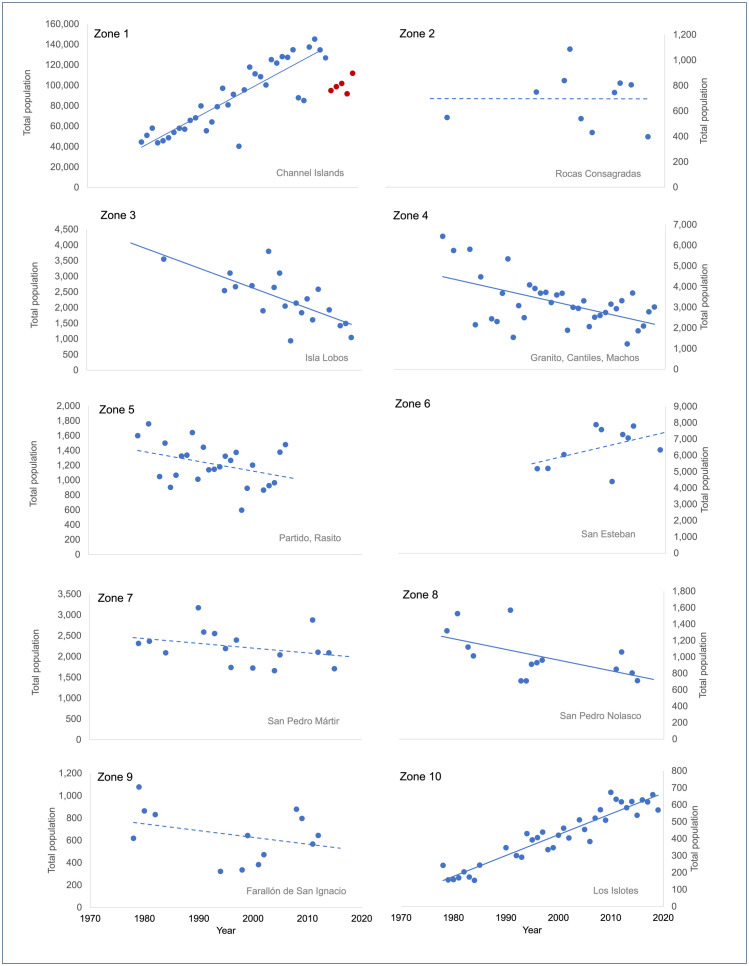
California sea lion population trends within Zones (1980–2020). Data shows total sea lion counts for each Zone with data from 1980–2020. Rookery names within each Zone are labeled in the bottom right of each panel. Zones composed of multiple rookeries show the sum of the population totals in those rookeries. Solid lines represent statistically significant regressions and dotted lines represent regressions that were not statistically significant. Red data points in Zone 1 represent population declines after the 2014 warming event (2015–2019); these data were not included in the overall regression.

This grouping of data from individual rookeries into composite geographic Zones was done for two main reasons: 1) to prevent potential over-representation of individual rookeries relatively close to each other that could be considered common ecological units, and 2) to best deal with the lack of available continuous population and diet data over time by grouping available data, thus allowing us to make comparisons between different geographic Zones for similar time periods and eras. While diet can change over a season and between rookeries on the same island (as is the case with Granito, Machos, and Cantiles) taking this into account was beyond the scope of the current study. Rookeries were therefore grouped into a common Zone if they occurred in a similar geographic area (<100 km away from each other) and had a similar population trajectory over time, which were determined by fitting a linear regression to the total population counts of each rookery for all years with available data (Fig 2). The average annual population change (percent) was calculated as the slope divided by the intercept (the predicted first year population) of the regression equation. Population trajectories were thereafter classified as increasing, decreasing, or inconclusive according to the slope of the linear regression.

Previous studies have partitioned the 13 Gulf of California rookeries into three sub-populations based on factors such as environmental conditions, genetic structure, and diet [14,37–40]. However, we deliberately excluded factors related to diet, and thus created Zones based only on similarities in population trajectory and geographic proximity.

Following this methodology, 10 Zones were created. The Channel Island rookeries were grouped together into Zone 1 (Fig 1), and the available data resulted in two Zone-era groupings: Zone 1 1981–1995 and Zone 1 2000–2011. The Gulf of California rookeries were grouped into 9 zones (Zones 2–10; Fig 1). Data for the Gulf of California using the available FO data resulted in two era groupings, 1990–2000 and 2015–2019. This resulted in 9 Zone-era groupings for the GoC using FO data. The IIMP data yielded three era groupings: 1995–1996, 2002, and 2015–2019 (S4 Table S1 File). This resulted in 18 Zone-era groupings for GoC. In all, this process yielded 11 Zone-era sets of matched population and FO-based diet quality data for the CI and GoC combined, and an additional 18 sets of data for the Gulf of California from IIMP-based diet quality. The relationship between diet quality and population change was then tested on these ‘Zone-era’ groupings.

### Effects of environmental change

Changes in diet quality before and after an event characterized by increased sea surface temperatures were explored by comparing the change in average diet energy density and diet diversity in the Gulf of California before and after 2014. Post-environmental shift diet data were not available for the Channel Islands (diet data available only up to 2011), so only pre-environmental shift data were used to compare the diet quality between the Gulf of California and the Channel Islands.

Prey taxa from the diet data were grouped into 9 species categories to further describe changes in diet before and after the environmental shift, and to express the ecological distribution of species consumed (number of prey taxa per category). These categories were assigned based on broad ecological characteristics similar to previous studies as per [41], and included: benthic species (n = 60 prey taxa), crustaceans (n = 1), gadids (n = 5), lanternfish (n = 7), octopus (n = 2), rockfish (n = 5), schooling fishes (n = 21), squids (n = 15), and miscellaneous (n = 17) (S5 Table in S1 File).

### Statistical analysis

To test relationships between population changes and diet quality, linear regression models were fit to the data in R-Studio (version 2022.02.3). Four separate simple linear regression models were used to test for relationships between population change and measures of diet diversity and energy density—from both MFO and MIIMP—at the level of ecological Zones using Zone-era data. To account for differences in population sizes among Zones, each Zone-era data point was weighted by an estimated Zone population size (reported in S4 Table in S1 File) calculated from the years used to assess population change. This provided a representative population size during the window surrounding each era and avoided relying on a single year’s value. All energy density and diet diversity values derived from MFO and MIIMP data were tested for outliers using Grubb’s and Dixon’s outlier tests in R-Studio using the package “outliers”, at the Zone-era grouping level. Preliminary analyses revealed no statistically significant outliers in the data.

Two-sample t-tests assuming unequal variances were conducted using MFO and MIIMP data to compare diet diversity and energy density within the Gulf of California before and after the 2014 environmental shift, and when comparing pre-environmental shift diets between the Gulf of California and the Channel Islands.

## Results

### Population trajectories

All four Channel Island rookeries (Zone 1) averaged population increases of 2–6% per year from 1980–2020, except for a population decrease associated with increased sea surface temperatures in 2014 (Fig 2). In contrast, the overall population in the Gulf of California decreased over the same time period, although trends differed at individual rookeries. While most rookery populations showed a decline, San Esteban and Los Islotes rookeries (Zones 6 and 10) showed increasing population trajectories (although the San Esteban population increase was not statistically significant). As opposed to the Channel Islands, sea lion populations in the Gulf of California did not seem to be collectively affected by the 2014 warming event (Fig 2).

### Diet quality and population changes

Diet diversity (using the Shannon Index) was not significantly related to rate of population change when examined at the level of Zone-eras (*p* = 0.43 using MFO data and 0.62, using MIIMP data; S3 Fig, left panels). Nor were there significant relationships between diet energy densities and rates of population change (*p* = 0.80 using MFO data and *p* = 0.13 using MIIMP data; S3 Fig, right panels).

### Diet quality in the Channel Islands vs. the Gulf of California

**Fig 3:**
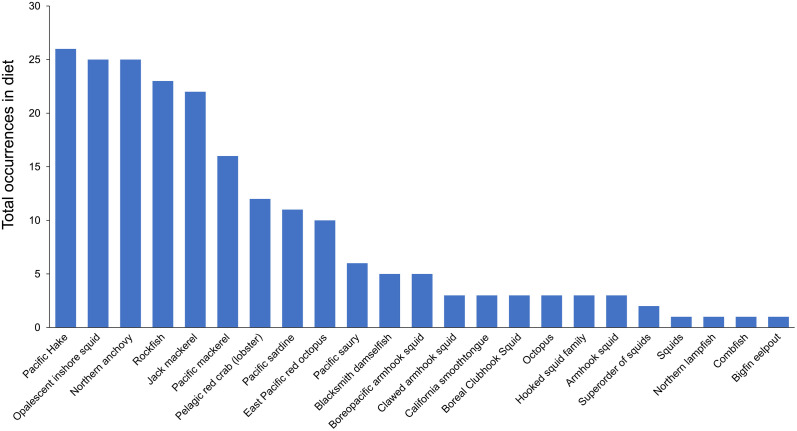
Prevalence of California sea lion prey taxa in the Channel Islands. Bars represent the total number of occurrences (out of 41 possible occurrences, equivalent to sample years) of each prey taxa from frequency of occurrence data from 1980–2011; that is, the total number of years where each prey taxa was present in the diet. All 23 prey taxa with FO ≥ 5% are listed. The prey taxa or species common name is listed when available, although some were identified only at the family or genus level.

**Fig 4:**
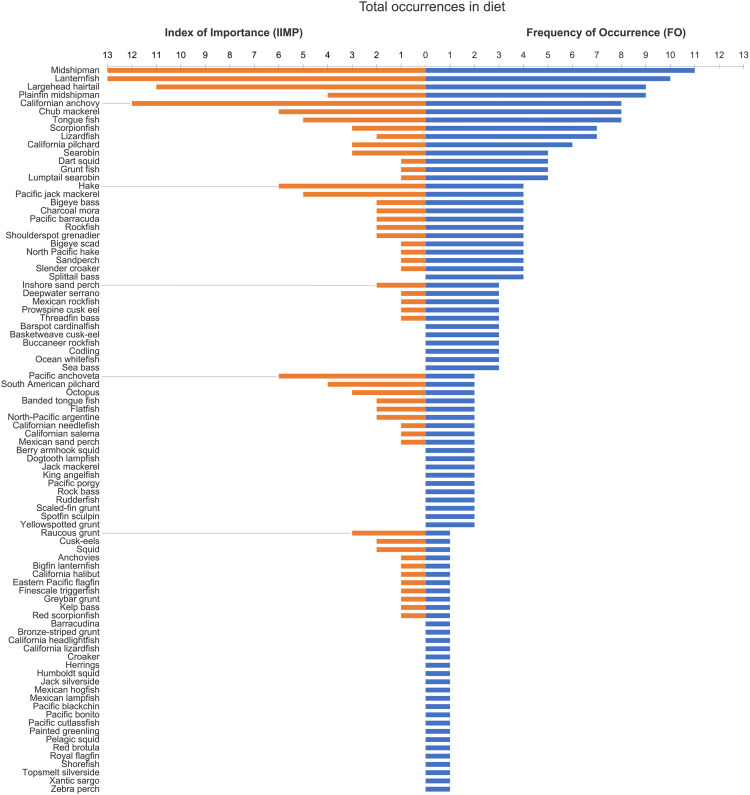
Prevalence of California sea lion prey taxa in the Gulf of California. Bars represent total number of occurrences (equivalent to years) of each prey taxa from available diet data from 1980–2019 for all Gulf of California rookeries (Zones 2–10) from either frequency of occurrence (blue bars) or index of importance (orange bars) data. All 88 prey taxa with FO or IIMP ≥5% are listed. The prey taxa common name is listed when available. The common name of all 88 main prey taxa (or species) from both FO and IIMP data are listed when available.

At the Channel Islands, California sea lions primarily consumed 23 main prey taxa, whereas they consumed 88 main prey taxa in the Gulf of California based on prey that had a mean frequency of occurrence (MFO) greater than 5% for each rookery and year (Figs 3 and 4). Jack mackerel, Chub mackerel, Pacific hake, and Californian anchovy coincided as top prey taxa consumed by sea lions in both the Channel Islands and the Gulf of California.

**Fig 5:**
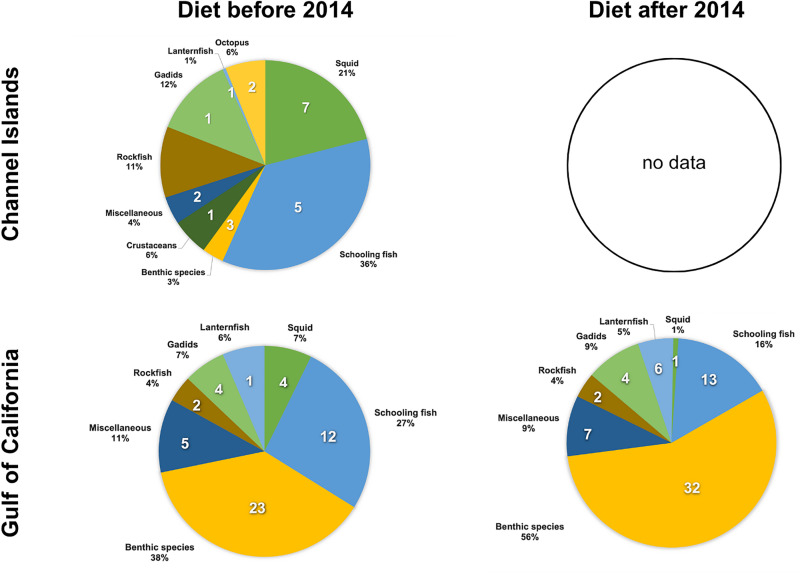
Diet composition by prey taxa categories before and after 2014 from frequency of occurrence data. Pie chart slices represent the proportion by each prey taxa category. White numbers represent the number of prey taxa in the diet from each category. Diet composition data from the Channel Islands is from 1980–2011 (no comparable diet data available after 2011). Diet composition data for the Gulf of California before the 2014 environmental shift is from 1990–2000 and after 2014 is from 2015–2019.

**Fig 6:**
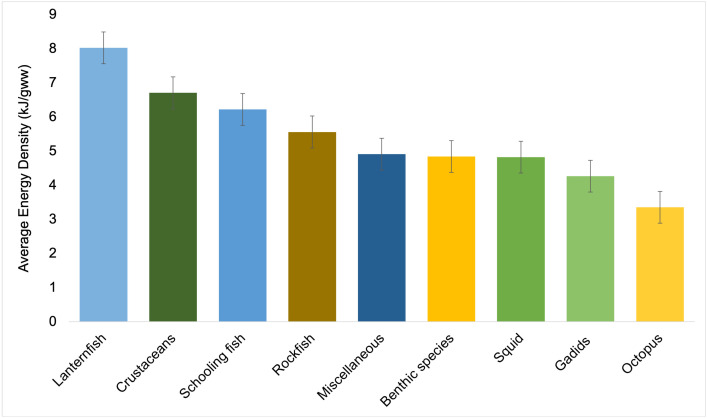
Average energy density of each prey taxa category consumed by California sea lions. Colours correspond to prey taxa categories illustrated in Fig 5. Bars represent average energy densities (kJ/gww; mean value ± standard error) from all prey taxa present in the diet data from each category ordered from highest (lanternfish) to lowest (octopus) value.

Sea lions in the Channel Islands mainly fed on schooling fishes (36%) and squid (21%) from 1981–2011. In comparison, sea lions in the Gulf of California consumed a diet dominated by benthic prey (41 prey taxa; 47% of diet) and fewer schooling fishes (19 prey taxa; 22% of diet; 1990–2019; Fig 5, left panels), with the schooling fishes having a higher average energy density than benthic species (Fig 6).

**Fig 7:**
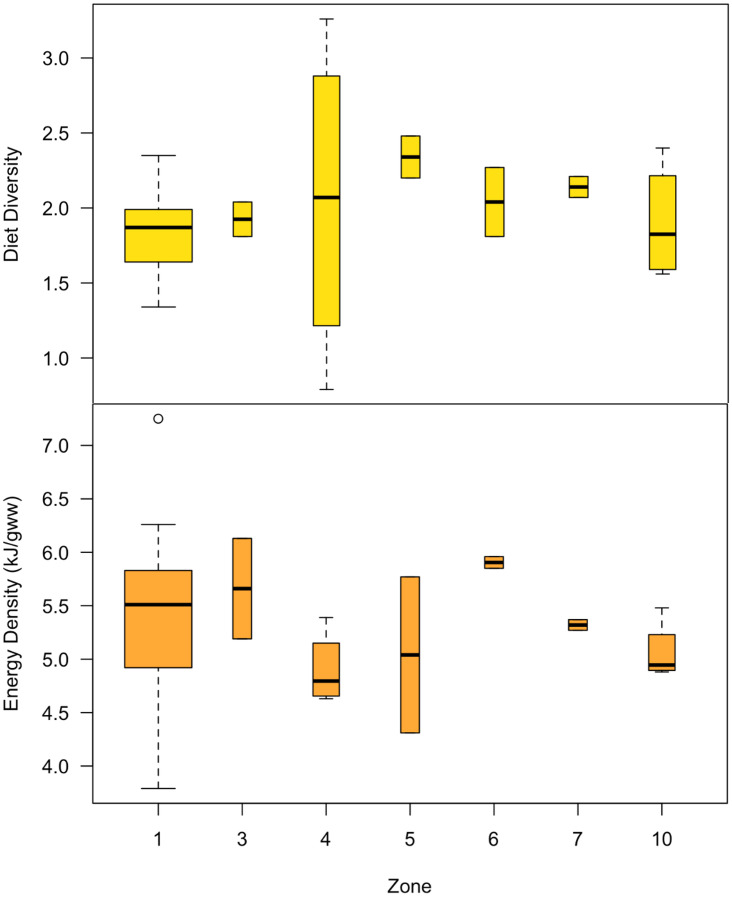
Average annual diet diversity and energy density by Zones from frequency of occurrence data. Diet diversity (top panel) and energy density (bottom panel) values were based on all available Zone and year groupings from MFO (1981–2019; Zones 2, 8 & 9 are omitted due to lack of available FO data). Diet diversity values were calculated using the Shannon Index. Box limits represent the first, second (mean), and third quantile values, and box whiskers represent the range of values. Bar widths are proportional to the number of data points in each Zone. The circle represents the energy density value of 7.25 in Zone 1 (San Miguel, 2005), which was considered an outlier according to Grubb’s test.

Mean diet energy density for sea lions in the Channel Islands (5.43 kJ/gww) was comparable to the Gulf of California (5.32 kJ/gww) (Table 1 and Fig 7). Mean diet diversity using the Shannon Index was lower in the Channel Islands than in the Gulf of California (1.83 vs. 2.04; Table 1 and Fig 7) and was more variable among the Gulf of California Zones (range: 0.79–3.26) than among the Channel Islands (range: 1.34–2.35). This higher variability was partly due to differences in diet diversity values using the Shannon Index between rookeries within the same Zone, but was predominantly due to differences in diet diversity values between eras. For example, diet diversity among the rookeries composing Zone 4 in the Gulf of California ranged from 0.90–1.96 in 1995–96, and 2.01–3.02 in 2016–2018 (S3 Table in S1 File).

**Table 1:** Long-term diet quality and population trajectory of each of the Gulf of California Zones and the Channel Islands from frequency of occurrence data.

Zone: rookery	Population trajectory	Energy density (kJ/gww)		Diet diversity (Shannon Index)		n
		Mean ±SD	Range	Mean ±SD	Range	
1: San Miguel	Increasing	5.43 ± 0.94	4.14–7.25	1.79 ± 0.94	1.34–2.35	10
1: San Nicolas	Increasing	5.63 ± 0.22	5.26–5.94	1.75 ± 0.23	1.46–1.96	8
1: San Clemente	Increasing	5.16 ± 0.84	3.79–6.05	1.94 ± 0.84	1.67–2.21	7
1: Santa Barbara	Increasing	5.48	N/A	1.87	N/A	1
**Channel Islands average**	**Increasing**	**5.43 ± 0.20**	**3.79–7.27**	**1.83 ± 0.12**	**1.34–2.35**	**4**
3: Isla Lobos	**Decreasing**	5.66 ± 0.66	5.19–6.13	1.93 ± 0.66	1.81–2.04	2
4: Machos, Cantiles, Granito	**Decreasing**	4.90 ± 0.35	4.63–5.39	2.05 ± 0.35	0.79–3.26	4
5: Rasito	Decreasing	5.04 ± 2.45	4.31–5.77	2.34 ± 1.03	2.20–2.48	2
6: San Esteban	Increasing	5.90 ± 2.83	5.85–5.96	2.04 ± 0.08	1.81–2.27	2
7: San Pedro Mártir	Decreasing	5.32 ± 2.50	5.27–5.37	2.14 ± 0.07	2.07–2.21	2
10: Los Islotes	**Increasing**	5.06 ± 2.17	4.88–5.48	1.90 ± 0.28	1.56–2.40	4
**Gulf of California average**	Decreasing	**5.32 ± 0.39**	**4.31–6.13**	**2.04 ± 0.15**	**0.79–3.26**	**6**

The mean and standard deviation of the range of the annual weighted diet energy density (kJ/gww) and of the diet diversity (Shannon Index) for sea lion diets is shown (incorporating individual prey taxa), as well as the number of years with data points (n) used to calculate the corresponding mean and range values. Long-term population trajectories for each rookery (1980–2020) are indicated with those in **bold** representing statistical significance. Zones 2, 8 & 9 are omitted due to lack of available FO data. Shorter term Zone-era population changes are provided in S4 Table in S1 File and S3 Fig.

The lowest diet diversity (0.79) occurred within Zone 4 (Granito, Cantiles, Machos) in 1996, which was heavily influenced by the Granito rookery where just one main species (FO ≥ 5%), largehead hairtail (*Trichiurus lepturus*)*,* was consumed that year. Interestingly, the highest diversity recorded among all locations (3.26) occurred in 2018 also at Granito where sea lions consumed a total of 31 main prey taxa (FO ≥ 5%). This was part of the general trend where diet diversity values from all three rookeries in Zone 4 in 2016 were also much higher than in 1996 (Table 1).

The mean diet energy density in the Channel Islands was 5.43 ± 0.2 kJ/gww, with a surprisingly small overall variation considering that two data points had anomalously high or low energy densities (7.25 kJ/gww in San Miguel, 2005; and 3.79 kJ/gww in San Clemente, 1982). The species that contributed the most to the average energy density in the diet primarily belonged to the schooling fishes category (Fig 6), and included jack mackerel, Pacific mackerel, and northern anchovy. San Miguel Island in particular had years when sea lion diets were above the calculated average energy density of 5.43 kJ/gww (S1 Fig) with a higher-than-normal contribution from two species of schooling fishes, Pacific sardine (2002–2005) and Pacific herring (2005), resulting in average energy densities up to 7.25 kJ/gww in 2005.

Although the overall diet energy density for sea lions in the Gulf of California was not statistically different than for those in the Channel Islands (Table 1; *p* > 0.05), there was significant variability between years and within Zones in the Gulf, reflecting differences in the prey taxa that contributed the most to the average energy density values. The highest diet energy density in any year within the Gulf occurred in Zone 3 (Isla Lobos, 1995, 6.13 kJ/gww), mainly due to the high energy density of Pacific anchoveta (a schooling fish) and largehead hairtail (a miscellaneous fish) (Table 1 and S2 Fig). However, diets in Zone 6 (San Esteban) had the highest mean energy density overall (5.90 kJ/gww; 1995 and 1996). For both Zones 6 and 7 (San Esteban and San Pedro Mártir), the overall energy density of the diet largely reflected a high contribution from lanternfish, followed by largehead hairtail, Californian anchovy, and chub mackerel (S2 Fig), species previously identified as dominant at these rookeries during those years [20]. Interestingly, in 1996 lanternfish was largely replaced with other prey taxa in the diet in both Zones 6 and 7 with little effect on the mean diet energy density. Diets of sea lions feeding in Zone 4 (Granito, Cantiles, Machos) had the lowest mean energy density (4.90 kJ/gww), which included a high proportion of ‘other’ prey taxa outside of the top 17 for most years (1996, 2016, 2018) (S2 Fig).

### Effect of environmental changes on diet quality in the Gulf of California

**Fig 8:**
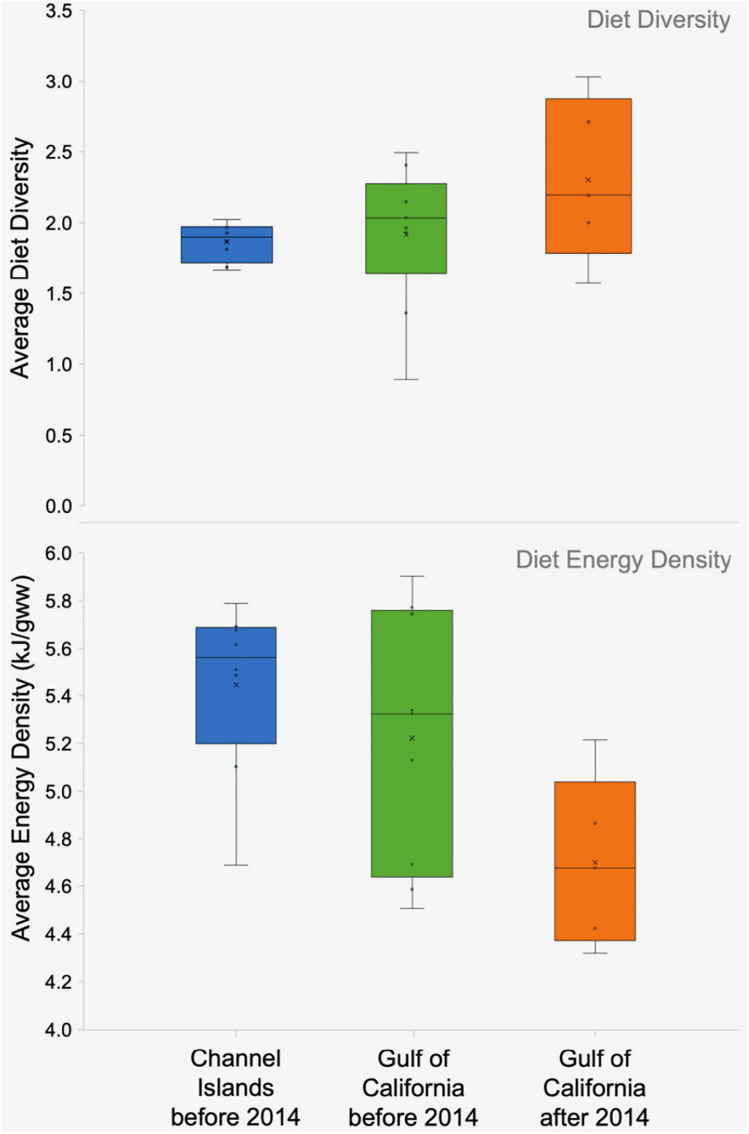
Average diet diversity from the Shannon Index (top panel) and average energy density (bottom panel) from frequency of occurrence data before and after 2014. Box limits represent averaged data (first, median and third quantiles ± standard error, ‘x’ represents mean value) from all Channel Island and Gulf of California groupings before and after 2014 (no data for Channel Islands after 2014). Diet data is based on rookery-year groupings. No significant differences were found between mean diversity values between geographic areas before 2014, nor in the Gulf of California between eras. No significant differences were found between mean energy density values between geographic areas before 2014, but there was a statistically significant decrease in the average energy density in the Gulf of California after 2014.

From 2014 to 2016, the Gulf of California experienced unusually high sea surface temperatures. During this period, significant declines occurred in the proportions of schooling fish (from 27% to 16%) and squid (from 7% to 1%) consumed by California sea lions, while the proportion of benthic prey taxa increased from 36% to 56% (Fig 5). These changes marked a shift from high-energy schooling fish to lower-energy-density benthic species, resulting in a significant reduction in the overall average energy density of their diet from 5.22 ± 0.53 to 4.69 ± 0.35 kJ/gww (two-tailed p = 0.045; Table 2, Fig 8, bottom panel).

**Table 2:** Diet quality by geographic area before and after 2014.

Geographic area & era	Number of main prey taxa in diet	Diet diversity (Shannon Index) (mean ±SD)	Energy density (kJ/gww; mean ±SD)
Channel Islands before 2014	23	1.83 ± 0.12	5.42 ± 0.36
Gulf of California before 2014	51	1.92 ± 0.49	5.22 ± 0.53
Gulf of California after 2014	65	2.30 ± 0.57	4.69 ± 0.35

Total number of diet main prey taxa, mean diet diversity using the Shannon Index, and mean weighted diet energy density.

Diet diversity calculated using the Shannon Index showed no significant difference in mean values in the Gulf of California before (1.92 ± 0.49) and after (2.30 ± 0.57) 2014 (two-tailed **p* *= 0.245; Table 2, Fig 8, top panel). However, there was an increase in the number of prey taxa consumed (from 51 to 65 taxa), and an overall increase in the average number of main prey taxa consumed per rookery after 2014. Before 2014, sea lions consumed 9 main prey taxa on average within rookeries (range 5–15 prey taxa), increasing to an average of 16 prey taxa per rookery (range 7–26 prey taxa) after 2014 (S4 Fig; data unavailable for Zones 8 and 9). Equally notable was that ~50% of the prey taxa consumed throughout the Gulf of California after 2014 were not present in the diet prior to this time (S5 Fig). In other words, the sea lions did not simply add 14 more prey taxa to their diet, but made a fundamental shift in the prey taxa they consumed. Despite the overall dietary shifts in prey quality observed after 2014, there were no apparent differences in the rates of population changes between these two eras (two-tailed **p* *= 0.984).

## Discussion

Previous dietary studies on California sea lions in the Gulf of California have tended to focus on detailing differences in the main prey taxa consumed, or describing feeding behaviours at various rookeries [20,21]. Only one study has indirectly explored the broad relationship between population changes and diet within the Gulf of California, finding no significant relationships between these variables [14]. Our study is the first to assess California sea lion diet quality using available long-term summer data on a finer geographic scale for the Gulf of California (1990–2019) and the Channel Islands (1980–2011), to explore its relationship to differences in population trajectories.

Our results demonstrate substantial differences in the diversity and type of main prey taxa consumed by sea lions in these two areas, but do not show any significant relationships between short-term measures of diet quality (diet energy density or diet diversity) and rates of population change. Results also demonstrate that following the significant increase in sea surface temperatures that occurred in 2014, sea lions in central regions of the Gulf of California consumed a greater number of prey taxa that had an overall lower diet energy density. However, this shift did not result in an overall population decline throughout the Gulf of California, as occurred in the Channel Islands. These findings underscore the importance of considering different aspects of environmental heterogeneity (e.g., prey availability, bathymetry, human impact) of different regions throughout the Gulf of California, which can heavily influence California sea lion population dynamics at local levels.

### The role of diet diversity

An ideal diet for California sea lions is one that allows them to meet their nutritional needs to grow and reproduce by feeding on sufficiently available prey taxa. However, our results illustrate that the exact nature of such a diet appears to vary depending on the characteristics of the ecosystem, making single indicators such as diet diversity difficult to interpret. Animals may choose to forage on fewer, energy-rich prey taxa (i.e., a low diversity, high energy density diet), which may reflect either a high degree of prey selectivity or a less biodiverse ecosystem. In more diverse ecosystems, foraging on a greater combination of species of different sizes and nutritional profiles might prove to be an optimal strategy and may even be required to fulfill nutritional requirements [42]. Different marine mammal species have exhibited switches to lower quality diets (lower energy density prey) during environmental challenges that have been characterized by either decreases [24,43] or increases [12,15,44] in diet diversity.

Using the total number of prey taxa as a measure of diet diversity in our study revealed a striking difference in diets between regions. The sea lions at the Channel Islands consistently consumed 23 primary prey taxa during the summer (1981–2011, Fig 3) while those in the Gulf of California consumed 88 primary prey taxa (1990–2019; Fig 4) that varied between Zones (S2 Fig). The lower number of prey taxa observed in the Channel Islands was in contrast to previous studies that found that prey species diversity (using the Shannon Index for species richness) are highest during summer [22]. These regional differences between the Channel Islands and the Gulf of California in the number of prey items may mean that the ideal prey species were not consistently available for sea lions in the Gulf, or it may alternatively illustrate that there were more prey options available to facilitate diet adaptability in some Zones.

Overall, the Shannon Index and the total number of prey taxa consumed illustrate that the diet has been relatively consistent over time within the Channel Islands (Fig 7, Table 1). In comparison, there was greater variability in diet composition in the Gulf of California both between Zones and over time within each Zone (S2 Fig). For example, the number of primary prey taxa consumed per rookery ranged from 5–26 prey taxa (S4 Fig), indicating greater resource heterogeneity and/or apparent dietary flexibility.

### The role of diet energy density

Regardless of its relationship to diet diversity, the energy density of a diet is an important characteristic to consider when assessing diet quality. As each prey taxa differs in macronutrient composition and therefore in energy density (kJ/gww), diets based upon prey taxa that are more energy-rich can be considered “higher quality” as they are more likely to meet the nutritional requirements of individuals, allowing populations to grow. Measuring diet energy density can provide important insight into the nutritional status of a population and the drivers of population change.

On average, the long-term energy density of diets across the Gulf of California did not differ significantly from that of the Channel Islands, such that energy density did not explain broad differences in population trajectories. Contrary to expectations, we found that regions and periods when the diet had the highest energy density were not necessarily associated with years of greatest population growth within Zones. Alternate analyses (i.e., using finer data at the rookery-year scale and using changes in pup numbers as a more immediate indicator of changes in population demographics) also did not reveal any relationships between diet energy density and population changes. Even the diets with the highest energy densities were associated with both increasing and decreasing population trajectories, indicative of the lack of an overall simple relationship between diets and populations over time.

Paradoxically, the highest mean diet energy density among all Zones—including the Channel Islands—was observed in the Gulf of California Zone 6 (San Esteban rookery), the largest rookery in the Gulf. However, this population only showed a potentially increasing trend despite its high-quality diet. Similarly, Zone 3 (Isla Lobos) had the second highest diet energy density but experienced a significantly decreasing population. In contrast, the only Zone in the Gulf with a significantly increasing population (Zone 10, Los Islotes rookery) had a mean diet energy density close to the median diet energy density across all Zones (Table 1). These patterns suggest that, despite evidence from other pinniped studies supporting a link between diet energy density and population growth [15,26], differences in diet quality in our study regions may not have been substantial enough to drive population trends, except perhaps in cases involving particularly low-quality diets such as that observed in the decreasing population in Zone 4. It is also likely that other factors––such as disease or human caused mortality––interact with diet and obscure any simple relationships that may exist between energy intake and population health.

An important factor to consider when exploring the relationship between populations and diet quality is that, as demonstrated by our results, the Channel Islands and the Gulf of California are fundamentally different oceanographic systems with different population and diet dynamics. In the Channel Islands, most of the diet energy density consistently comes from schooling fishes (S1 Fig). In comparison, there is considerable variability in the diet of sea lions in the Gulf of California, which is made up of different combinations of benthic organisms (41 different main prey taxa in total) making up their main source of energy from food (S2 Fig), even though this prey category has a lower average energy density than schooling fishes. In fact, the greater predictability of the prey available to sea lions in the Channel Islands than those in the Gulf of California may be a more important contributor to population dynamics, rather than differences in prey energy density per se.

The heterogeneity in diet between rookeries throughout the Gulf of California raises questions about the specific trade-offs and foraging strategies among sea lions breeding at different rookeries. In the Galápagos Islands, individual foraging strategies of Galápagos sea lions (*Zalophus wollebaeki*) influence the coping abilities of their population with evidence that some foraging strategies may be more advantageous than others during environmental changes [45]. In contrast to pelagic foragers, benthic foraging Galápagos sea lions appear to be less affected by increased water temperatures, despite consuming prey that are lower in energy density. Such environmentally dependent fitness trade-offs could also be at play in the Gulf of California sea lion populations, which should be explored through further research on individual foraging strategies.

### The effects of environmental changes on diet quality

From 2013–2015, a large-scale phenomenon of increased sea surface temperatures known as “The Blob” was first documented in Alaska and then traveled south along the Eastern Pacific. In 2015–2016, The Blob coincided with a strong El Niño event, further intensifying the effect of increased water temperatures [46]. In the Channel Islands, El Niño events are known to cause population declines in California sea lions (with rapid subsequent recovery), and are associated with a decrease in consumption of energy-rich schooling fish [17]. The Blob’s effects on the Channel Island populations were first seen in 2013 through increased pup mortality [22].

Unlike the Pacific coast, sea lions within the Gulf of California are not affected in the same way by warming events in the Pacific Ocean, such as El Niño. Instead, they appear to be affected by local oceanographic processes within the Gulf’s sub-regions [5]. Overall, although the increased water temperatures in the Gulf after 2014 were characterized by an increase in the mean and range of diet diversity within the Gulf of California (expressed using the Shannon Index), this change was not significant (Fig 8, top panel). However, there was an increase in the total number of prey taxa present in the diet (from 51 to 65 prey taxa, Table 2). In addition to the increase in the number of prey taxa consumed, the proportion of each diet prey taxa category changed (Fig 5), as well as the identity of the species within those categories. For example, around 50% of the prey taxa consumed after 2014 were not present in the diet in previous years. These prey taxa consisted mostly of new benthic and lanternfish species (Figs 5 and S5).

**Fig 9:**
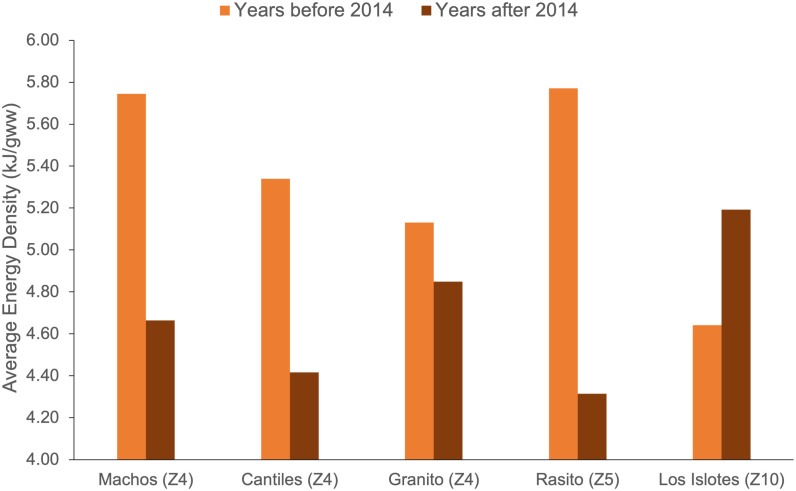
Average energy density of California sea lion diets at rookeries in the Gulf of California before and after 2014 from frequency of occurrence data. Only rookeries within Zones with matched data before and after 2014 are included in this Figure, with their Zone number is shown in brackets. Gulf of California Zones are ordered geographically from North (Z4) to South (Z10). Zones that are not shown here lacked data after 2014 and were excluded from this Figure.

The increase in diet diversity we noted was accompanied by a significant decrease in the overall average diet energy density throughout the Gulf of California (Fig 8, bottom panel). This trend was driven by central rookeries (Zones 4 and 5) where there was a decrease in the proportion of energy-rich schooling fishes after 2014 (Fig 9). Although there was also an increase after 2014 in the overall number of high-energy lanternfish species in the diet (from 1 to 6 prey taxa; Fig 5), there was no significant change in their proportion in the diet (6% vs. 5%). This suggests that sea lions may have attempted to continue to meet their total energy requirements by increasing the diversity of energy-rich lanternfish species in their diet.

Although a general decline in diet energy density occurred after 2014—mainly due to a decrease in energy-rich schooling fishes and lanternfish—this trend was not consistent across all rookeries. For example, diet energy density *decreased* at Rasito (Zone 5) after 2014, which lacked energy-rich lanternfish and Jack mackerel in 2016 compared to 1996 (S2 Fig). Conversely, within Zone 10 (Los Islotes rookery), an increase in lanternfish in 2019 was largely responsible for the overall *increase* in diet energy density after 2014 (Fig 9).

The observed differences in the changes in diet energy density post-2014 among different Zones could be due to a difference in the availability of prey taxa in the various regions in the Gulf of California. In some Zones this could lead to California sea lions having to adopt atypical, lower-energy diets. For example, the unusual dominance of ‘other’ prey taxa in the diet in Los Islotes in 2015 may reflect a loss of ideal primary prey due to increased water temperatures (S2 Fig), resulting in them consuming a higher number of prey taxa (S4 Fig) to maintain the same level of dietary energy density from prey (~5kJ/gww).

Previous studies have demonstrated how acute environmental changes and subsequent prey availability shifts can affect marine mammal population growth. For example, ringed seals (*Phoca hispida*) in western Hudson Bay switched to a more diverse diet that had a lower energy density due to decreases in the availability of their main prey (sand lance) triggered by changes in the seasonal breaking up of sea ice [15]. As a result, body condition of individual seals was greatly reduced, and population declines ensued.

In the Channel Islands, models predict that every 1°C increase in surface temperature could decrease the population growth rate of California sea lions in the U.S. by 7% [47]. Of note, during years of increased sea surface temperatures (2013–2015), sea lion diet composition in the Channel Islands decreased in epipelagic species (schooling fish), and increased in benthic and demersal species [22]. A similar phenomenon appears to have occurred in the northern and central regions of the Gulf of California where California sea lion pup birth rates declined as sea surface temperature anomalies exceeded 1°C (1978–2018) [14]. However, population growth in the southern regions of the Gulf was not affected by increased sea surface temperatures [48], although pup abundance and body condition at Los Islotes did decrease during 2014 and 2015, and adult females were away from the rookery for longer periods than normal [49]. Lactating females appeared to have had to forage further away from the rookery, which cost them more time and energy. Thus, warmer sea surface temperatures affected both the diet and foraging behaviour of female California sea lions in the south (Los Islotes, Zone 10), which may have affected pups during the lactation period.

### Environmental heterogeneity and its implications for species management

The variable diet quality and population trends detailed in our study suggest that sea lions at different rookeries, even those just within the Gulf of California, cannot be viewed nor managed as a homogeneous group. The Gulf of California is known to have considerable environmental heterogeneity [50–53], which may influence both the quality of sea lion diets as seen in our study and, ultimately, predator-prey dynamics at the rookery scale. It has been suggested, for example, that compared to the northern and central regions of the Gulf, the greater diversity of prey taxa present in the south may buffer rookeries like Los Islotes against detrimental environmental changes [53,54]. Having access to a greater diversity of prey taxa would allow sea lions to compensate for prey that may no longer be available.

Prey availability and abundance in the Gulf of California varies by region and is not as consistent or as predictable as in the California Current System. Such variability may mask the ability to identify simple relationships between diet and population growth, such as those shown in other Eastern Pacific ecosystems like the Channel Islands or Alaska [25,35,41]. This variability may also underly differences noted by others in terms of genetic differences between California sea lions, their foraging areas, and the oceanographic conditions they experience [4,14,38]. Understanding diet and population dynamics in the Gulf of California may therefore require a more detailed understanding of sea lion prey dynamics, foraging strategies, and localized oceanographic changes.

The Mexican government deems the California sea lion sub-populations in need of special protection, and recognizes the need to recover and conserve the populations in Mexico’s rookeries [55]. Our study highlights the variation in the diets, population trajectories, rookery sizes, and oceanographic dynamics within the Gulf of California, suggesting that each rookery population faces different sets of challenges that impact their reproduction and survival rates in different ways.

However, current management and surveillance programs (especially in the central Gulf of California) appear insufficient to monitor and assess how sea lion numbers are being affected. More rigorous data and monitoring is needed not only to track changes in prey taxa, but also assess other short-term factors affecting sea lion numbers such as entanglements in fishing gear, shootings, disease, and contaminants, to name a few [34,56]. Such anthropogenic factors may also contribute to the lack of a direct relationship between diet and population trends in the northern and central Gulf of California [14]. Overall, understanding the complex dynamics affecting each sub-population in both the short-term and long-term is essential to effectively manage the protection and conservation of California sea lions in the Gulf of California.

## Supporting information

S1 Methods Supplementary Methods. (DOCX)

S1 Fig Energy density of California sea lion diets in the Channel Islands (Zone 1). Average energy density and energetic content contributions (average weighted energy density) of the top 17 prey taxa to the total energetic content of the diet for rookeries and years with available frequency of occurrence data in the Channel Islands. ‘Other’ category represents all other taxa in the diet beyond the top 17.
(TIF)

S2 Fig Energy density of California sea lion diets in the Gulf of California (Zones 3–7, 10). Average energy density and energetic content contributions (average weighted energy density) of the top 17 prey taxa to the total energetic content of the diet for rookeries and years with available frequency of occurrence data in the Gulf of California. ‘Other’ category represents all other taxa in the diet beyond the top 17.
(TIF)

S3 Fig Population changes and diet quality for California sea lions from frequency of occurrence and index of importance data. The data represents values from Zone-era groupings. Diet diversity values were calculated using the Shannon Index from frequency of occurrence data (top-left panel) and index of importance data (bottom-left panel). Panels on the right include diet energy density values calculated using frequency of occurrence (top) and index of importance data (bottom). Regression analysis of data weighted by rookery population size indicated no statistical relationships.
(TIF)

S4 Fig Average number of main prey taxa per California sea lion rookery before and after 2014 from frequency of occurrence data. Blue bars represent each of the Channel Islands before 2014, each rookery with available data in the Gulf of California is shown before 2014 (green bars) and after 2014 (orange bars). Zone number is shown in brackets.
(TIF)

S5 Fig Total number of prey taxa consumed by California sea lions in the Gulf of California before and after 2014 from frequency of occurrence data. The bar on the left represents prey taxa before 2014, the bar on the right represents prey taxa after 2014. Green bars show number of prey taxa present in both eras, yellow bar shows taxa only present before 2014, and orange bar shows number of prey taxa present only after 2014.
(TIF)

S1 File **S1 Table**. Population data sources for California sea lions in the Channel Islands and the Gulf of California (1980–2020) used in this analysis. **S2 Table**. Diet data sources for California sea lions in the Channel Islands and the Gulf of California (1980–2020) used in this analysis. **S3 Table**. Raw paired diet and population data for California sea lions by rookery-year grouping. **S4 Table**. Raw paired diet and population data for California sea lions by Zone-era grouping. **S5 Table**. List of 114 diet prey taxa (scientific and common name) consumed by California sea lions in the Gulf of California (MEX) or the Channel Islands (USA) showing their average energy density, category assigned, and country where data was collected.
(DOCX)
