# Supplementary material for: Environmental heterogeneity plays a bigger role than diet quality in driving divergent California sea lion population trends
Source: PLoS One. 2025 Nov 5;20(11):e0324108. doi: 10.1371/journal.pone.0324108 (PMC12588526; doi:10.1371/journal.pone.0324108)
Supplement: S1 Methods — (DOCX) [file pone.0324108.s001.docx]

**S1 Methods. Supplementary Methods**

*Calculating population change rules*

In instances where a rookery had diet data for 3 or more continuous years, population data from one year before to one year after the diet data years were incorporated into the population change calculation. If the number of continuous years with diet data was less than three, then the population change was calculated from two years before and after the interval (or single year) of diet data. This yielded a single rate of population change over the matching diet data interval. In cases where population changes using the previously mentioned rules were calculated to be greater than ±20% (an unrealistic growth rate under normal breeding conditions), four years on either side of the diet data were incorporated into the calculation to obtain a more realistic rate of population change.

### Estimating missing population totals

Rookeries and years with available diet data were paired with available population totals. In some cases, population totals had to be estimated for years that lacked data. For the Channel Island rookeries, pup counts were available for years with missing population totals, and were therefore used to estimate totals by extrapolating from the linear relationship between pup counts and total population counts across all years. For the Gulf of California rookeries, missing population numbers were estimated by extrapolating from a linear regression performed on all available population data for individual rookeries because additional years with pup count data were not available. The regression equation was then used to estimate population numbers for years lacking counts, and the rate of population change over the period of interest was calculated from this mixed data set. Inaccurate extrapolations were avoided by only using regressions that spanned intervals with actual counts.

In cases where rookeries had one year of diet data and where the associated population data range (when incorporating the ± 2 years rule for population change) overlapped with the population data range for another year of diet data, *and* one of those diet data years did not have +2 years of data after (due to it being the latest population year with data), those years of diet data were grouped. For example, diet data from the Granito rookery from 2016 and 2018 were combined into one grouping, and the years 2014–2018 were used to calculate population change since 2018 was the latest year with population data.

*Calculating rookery-year groupings*

We matched the available diet data at the rookery level with a rate of population change value calculated to correspond to the specific year or group of consecutive years with available diet data. Ideally, continuous diet data for all years and rookeries would have been available, and matching data groupings would have been strategically chosen. However, diet data were patchy in terms of both years and rookeries. As a result, sequential data points were compiled from each rookery to form specific ‘year-rookery groupings’ and were treated as independent data points used in all subsequent analyses (Table S2).

Most rookeries in the Channel Islands had diet data available over several consecutive years, which were grouped according to the continuity of the data (e.g., San Miguel 2009–2011). Diet data from several rookeries in the Channel Islands were already averaged over several years (Table S2). In these instances, those year-rookery groupings were kept and used when calculating corresponding population changes. Within the Gulf of California, most years with diet data were single isolated years that varied by rookery (e.g., Los Islotes 1990, 2000, 2015 and 2019; Rasito 1996 and 2016, etc.). In cases where two consecutive years of diet data were available, we averaged the diet data and grouped them to form one rookery-year grouping (e.g., San Esteban 1995–1996). In cases where non-continuous years with diet data were close in time such that their population change calculations overlapped, we also combined them to form a single rookery-year grouping (e.g., Los Islotes 2015, 2019).
