# Supplementary figures and images for: Environmental heterogeneity plays a bigger role than diet quality in driving divergent California sea lion population trends
Source: PLoS One. 2025 Nov 5;20(11):e0324108. doi: 10.1371/journal.pone.0324108 (PMC12588526; doi:10.1371/journal.pone.0324108)

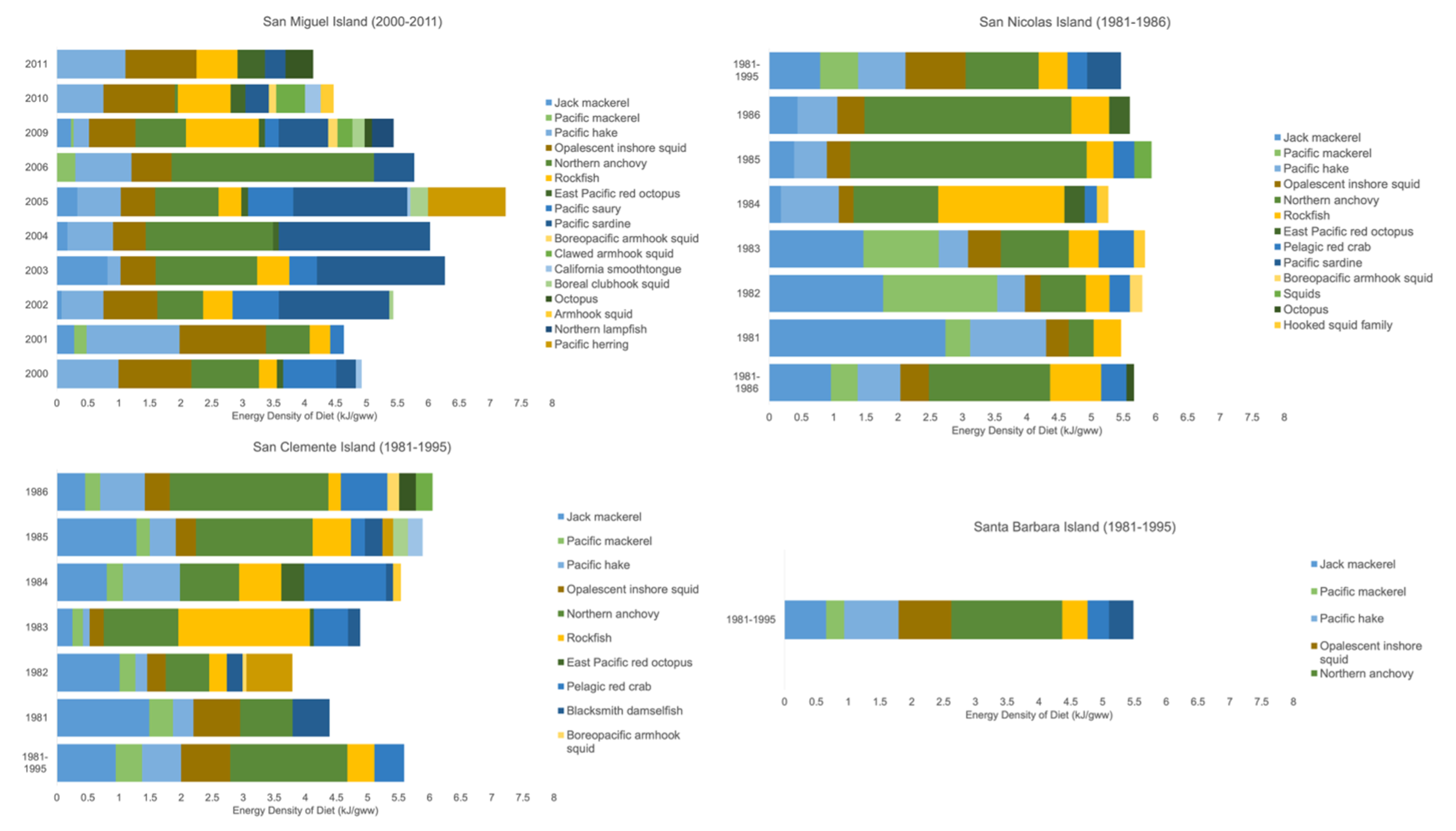

Supplement: S1 Fig — Average energy density and energetic content contributions (average weighted energy density) of the top 17 prey taxa to the total energetic content of the diet for rookeries and years with available frequency of occurrence data in the Channel Islands. ‘Other’ category represents all other taxa in the diet beyond the top 17. (TIF) [file pone.0324108.s002.tif]

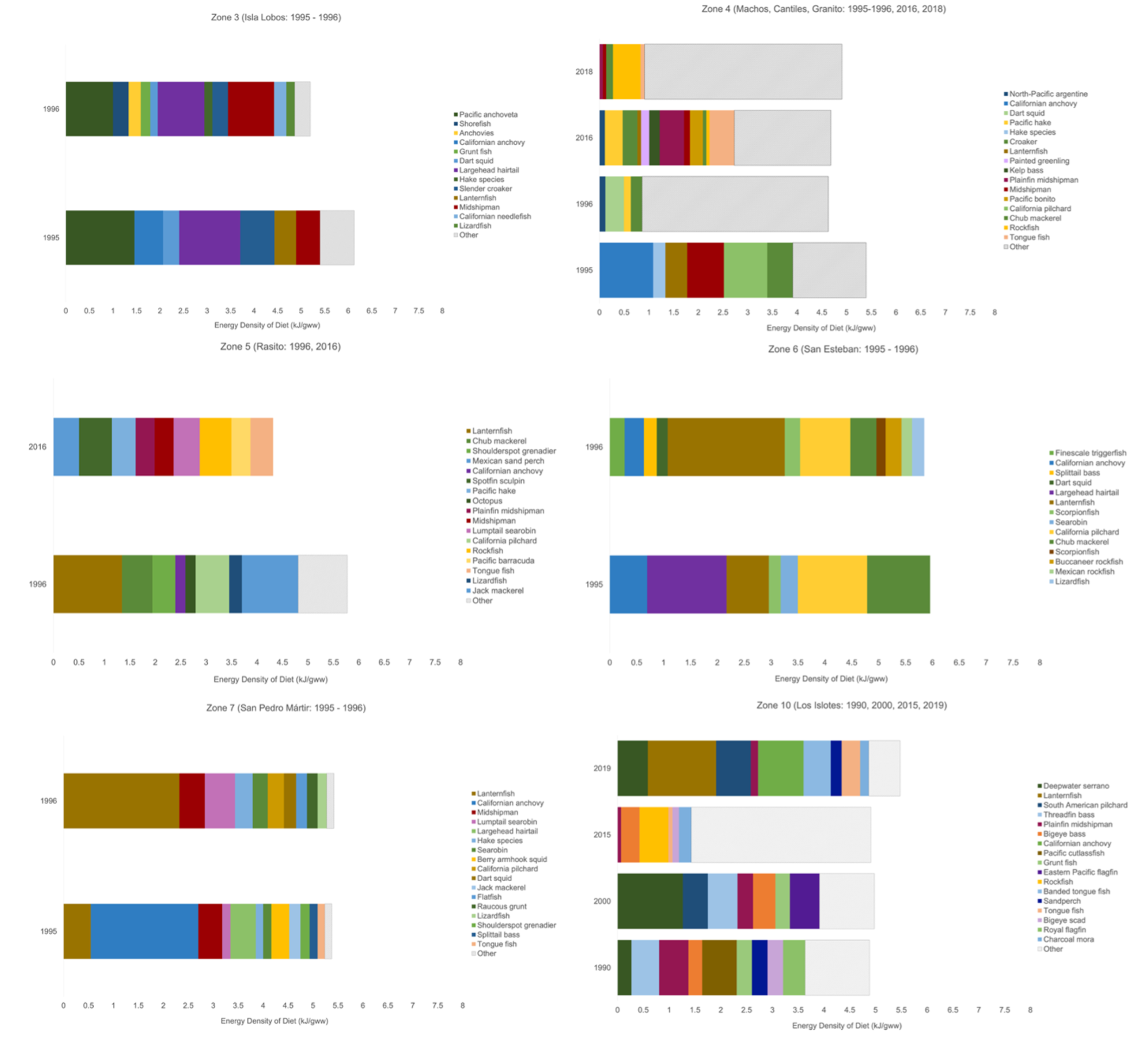

Supplement: S2 Fig — Average energy density and energetic content contributions (average weighted energy density) of the top 17 prey taxa to the total energetic content of the diet for rookeries and years with available frequency of occurrence data in the Gulf of California. ‘Other’ category represents all other taxa in the diet beyond the top 17. (TIF) [file pone.0324108.s003.tif]

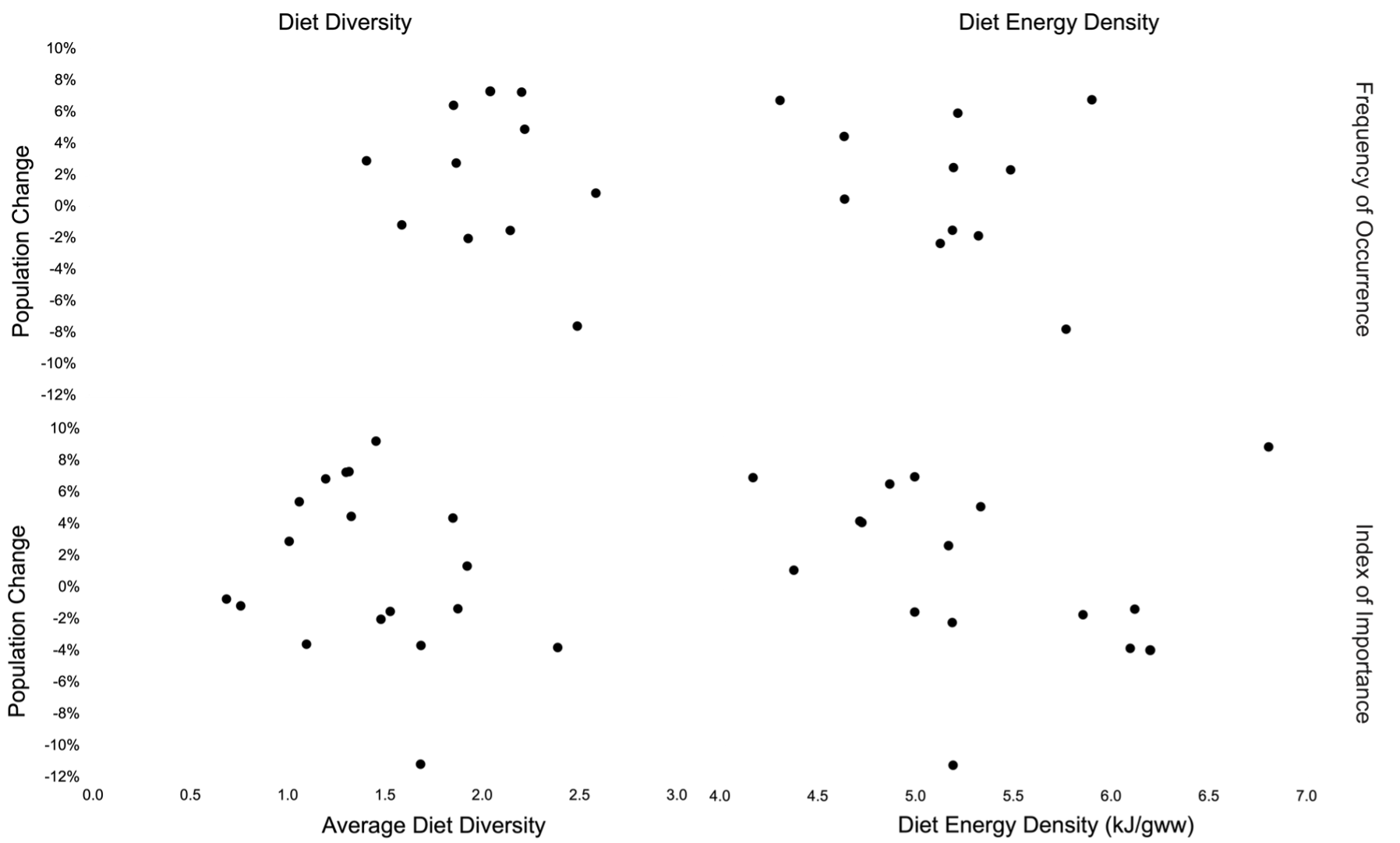

Supplement: S3 Fig — The data represents values from Zone-era groupings. Diet diversity values were calculated using the Shannon Index from frequency of occurrence data (top-left panel) and index of importance data (bottom-left panel). Panels on the right include diet energy density values calculated using frequency of occurrence (top) and index of importance data (bottom). Regression analysis of data weighted by rookery population size indicated no statistical relationships. (TIF) [file pone.0324108.s004.tif]

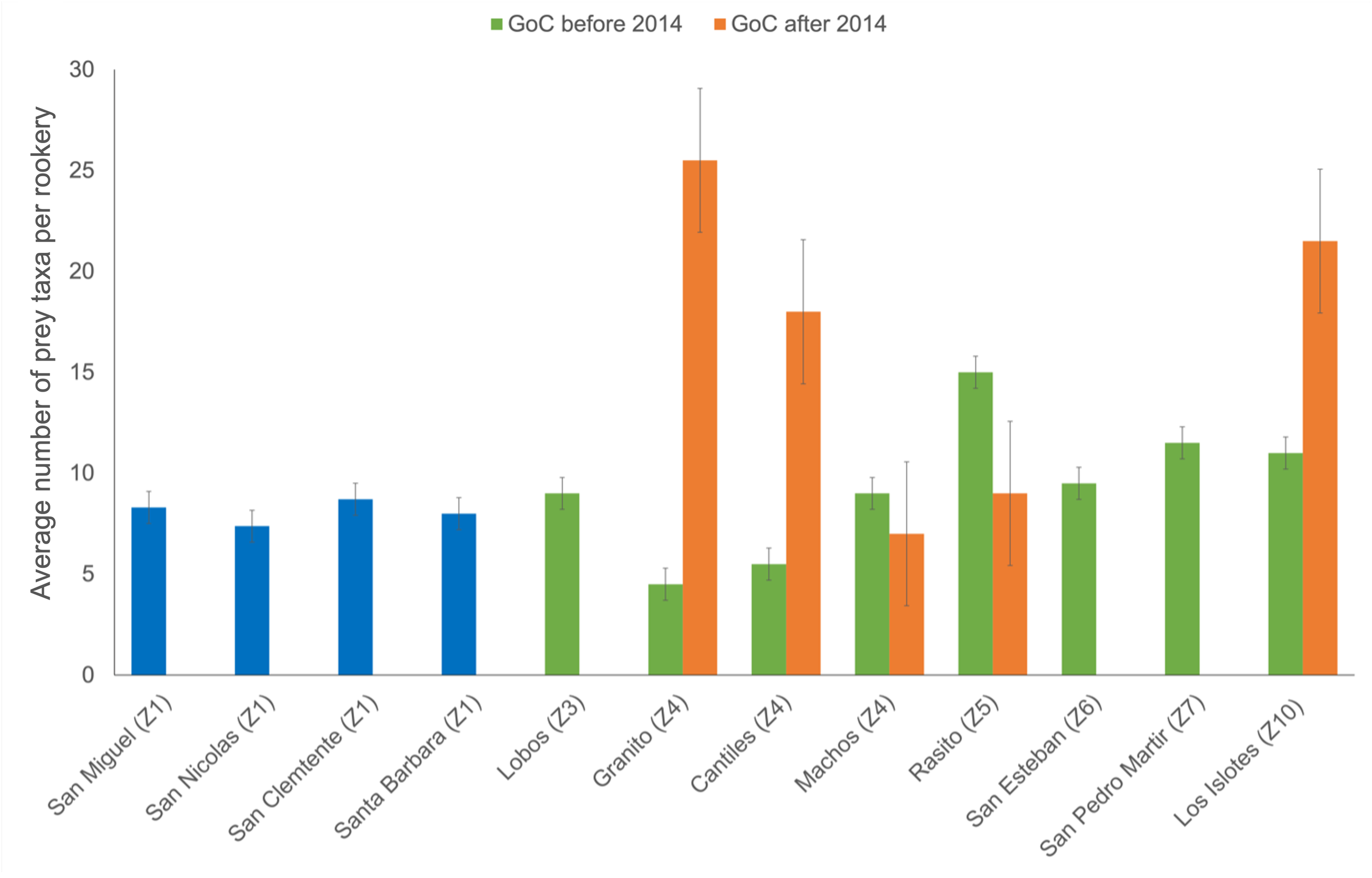

Supplement: S4 Fig — Blue bars represent each of the Channel Islands before 2014, each rookery with available data in the Gulf of California is shown before 2014 (green bars) and after 2014 (orange bars). Zone number is shown in brackets. (TIF) [file pone.0324108.s005.tif]

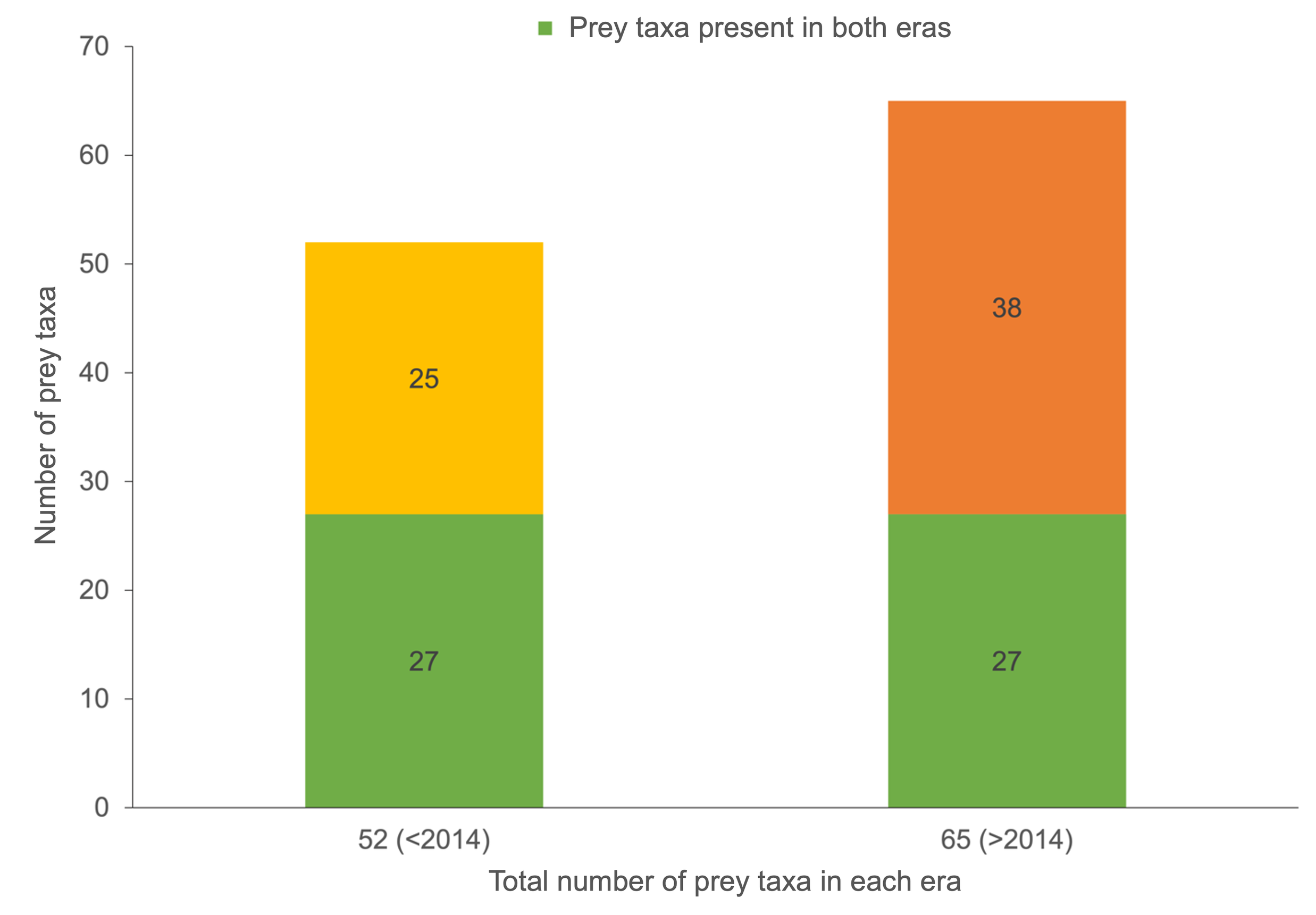

Supplement: S5 Fig — The bar on the left represents prey taxa before 2014, the bar on the right represents prey taxa after 2014. Green bars show number of prey taxa present in both eras, yellow bar shows taxa only present before 2014, and orange bar shows number of prey taxa present only after 2014. (TIF) [file pone.0324108.s006.tif]
