# Supplementary material for: Environmental heterogeneity plays a bigger role than diet quality in driving divergent California sea lion population trends
Source: PLoS One. 2025 Nov 5;20(11):e0324108. doi: 10.1371/journal.pone.0324108 (PMC12588526; doi:10.1371/journal.pone.0324108)
Supplement: S1 File — S1 Table. Population data sources for California sea lions in the Channel Islands and the Gulf of California (1980–2020) used in this analysis. S2 Table. Diet data sources for California sea lions in the Channel Islands and the Gulf of California (1980–2020) used in this analysis. S3 Table. Raw paired diet and population data for California sea lions by rookery-year grouping. S4 Table. Raw paired diet and population data for California sea lions by Zone-era grouping. S5 Table. List of 114 diet prey taxa (scientific and common name) consumed by California sea lions in the Gulf of California (MEX) or the Channel Islands (USA) showing their average energy density, category assigned, and country where data was collected. (DOCX) [file pone.0324108.s007.docx]

**S1 Table. Population data sources for California sea lions in the Channel Islands and the Gulf of California (1980–2020) used in this analysis.**

| **Rookery** | **Year** | **Season** | **Source** |
| --- | --- | --- | --- |
| San Miguel | 1971–1991 (pup counts only) | July or August | Lowry et al. (2017a) |
|  | 1992–2014 | July or August | Lowry et al. (2017a) |
| San Nicolas | 1991–2008 | July | Lowry et al. (2017a) |
|  | 2009, 2010 (pup counts only) | July | Lowry et al. (2017a) |
|  | 2011–2014 | July | Lowry et al. (2017a) |
| San Clemente | 1981–2014 | July or August | Lowry et al. (2017a) |
| Santa Barbara | 1983–1985 (pup counts only) | July | Lowry et al. (2017a) |
|  | 1986–2008 | July | Lowry et al. (2017a) |
|  | 2009, 2010 (pup counts only) | July | Lowry et al. (2017a) |
|  | 2011–2014 | July | Lowry et al. (2017a) |
| Channel Islands (grouped) | 2015 | July | Lowry et al. (2017b) |
|  | 2016-2019 | July or August | Lowry et al. (2021) |
| a-q (not all rookeries have data for all years) | 1980-2019 | June or July | Pelayo-González et al., (2021) |

### Details include the rookery, year and season of data collection, and the source publication from which the data were obtained. “Pup counts only” were used to extrapolate to total counts, see section “Estimating missing population totals” in Supplementary Methods.

### References for citations in “Source” column are the following:

- Lowry et al., 2017a: Lowry M, Melin S, Laake J. Breeding season distribution and population growth of California sea lions, Zalophus californianus, in the United States during 1964-2014. NOAA-TM-NMFS-SWFSC-574. U.S. Department of Commerce. 2017.
- Lowry et al., 2017b: Lowry M, Nehasil SE, Jaime EM. Distribution of California sea lions, Northern elephant seals, Pacific harbor seals, and Steller sea lions at the Channel Islands during July 2011-2015. National Oceanic and Atmospheric Administration. 2017. https://doi.org/10.7289/V5/TM-SWFSC-578
- Lowry et al., 2021: Lowry M, Jaime EM, Moore JE. Abundance and distribution of pinnipeds at the Channel Islands in Southern California, Central and Northern California, and Southern Oregon during summer 2016-2019. NOAA-TM-NMFS-SWFSC-656. National Oceanic and Atmospheric Administration. 2021. https://doi.org/10.25923/6qhf-0z55
- Pelayo-González et al., 2021: Pelayo-González L, Aurioles-Gamboa D, Godínez-Reyes C, Rubio-Rodríguez U, Hernández-Camacho CJ, Gallo-Reynoso JP, et al. Effect of environmental variables on the number of births at California sea lion (*Zalophus californianus*) rookeries throughout the Gulf of California, Mexico. Aquat Conserv Mar Freshw Ecosyst. 2021; 1–19. doi:10.1002/aqc.3545

**S2 Table. Diet data sources for California sea lions in the Channel Islands and the Gulf of California (1980–2020) used in this analysis.**

| **Rookery (letter, Zone)** | **Year (n = scat samples with hard parts)** | **Season** | **Source** |
| --- | --- | --- | --- |
| **Frequency of Occurrence (FO) data** | | | |
| San Miguel (a, 1) | 2000 (63)  2001 (61)  2002 (86)  2004 (91)  2005 (86)  2009 (44) | July–early August | Melin et al. (2010) |
|  | 2000 (154)  2001 (61)  2002 (98)  2003 (96)  2005 (62)  2009 (64)  2010 (57)  2011 (44) | June–Sept | Melin et al. (2012) |
|  | 2002 (14)  2003 (72)  2004 (56)  2005 (6)  2006 (27) | March–July | Orr et al. (2011) |
| San Nicolas (b, 1) | 1981 (24)  1982 (68)  1983 (150)  1984 (107)  1985 (64)  1986 (80) | June-August | Lowry et al., (1991)* |
|  | 1981–1995 (2,980; grouped) | January, April, July, October | Lowry and Carretta (1999) |
| Santa Barbara (c, 1) | 1981–1995 (736; grouped) | July | Lowry and Carretta (1999) |
| San Clemente (d, 1) | 1981 (48)  1982 (150)  1983 (87)  1984 (89)  1985 (60)  1986 (65) | June-August | Lowry et al., (1990)* |
|  | 1981–1995 (2,543; grouped) | January, April, July, October | Lowry and Carretta (1999) |
| Lobos (g, 3) | 1995 (27) | June | Data provided by co-author F.G-R |
|  | 1996 (16) | May | Data provided by co-author F.G-R |
| Granito (h, 4) | 1995 (12) | June | Data provided by co-author F.G-R |
|  | 1996 (7) | May | Data provided by co-author F.G-R |
|  | 2016 (10) | October | Pelayo-González et al., (2021) |
|  | 2018 (19) | August | Pelayo-González et al., (2021) |
| Cantiles (i, 4) | 1995 (7) | June | Data provided by co-author F.G-R |
|  | 1996 (11) | May | Data provided by co-author F.G-R |
|  | 2016 (8) | *Unknown* | Pelayo-González et al., (2021) |
|  | 2018 (20) | July | Pelayo-González et al., (2021) |
| Machos (j, 4) | 1995 (20) | June | Data provided by co-author F.G-R |
|  | 2016 (7) | October | Pelayo-González et al., (2021) |
| Rasito (l, 5) | 1995 (9) | June | Data provided by co-author F.G-R |
|  | 1996 (15) | May | Data provided by co-author F.G-R |
|  | 2016 (2) | October | Pelayo-González et al., (2021) |
| San Esteban (m, 6) | 1995 (24) | June | Data provided by co-author F.G-R |
|  | 1996 (35) | May | Data provided by co-author F.G-R |
| San Pedro Mártir (n, 7) | 1995 (15) | June | Data provided by co-author F.G-R |
|  | 1996 (26) | May | Data provided by co-author F.G-R |
| Los Islotes (q, 10) | 2000-2001 (164; grouped) | May to April | Cardenas-Palomo (2003) |
|  | 2015 (12) | July | Pelayo-González et al., (2021) |
|  | 2019 (12) | August | Pelayo-González et al., (2021) |
| **Index of Importance (IIMP) data** | | | |
| Rocas Consagradas (e, 2) | 2002 (21) | July | Porras-Peters (2004) |
| Lobos (g, 3) | 1995 (27) | June | Data provided by co-author F.G-R |
|  | 1996 (16) | May | Data provided by co-author F.G-R |
|  | 2002 (9) | July | Porras-Peters (2004) |
| Granito (h, 4) | 1995 (12) | June | Data provided by co-author F.G-R |
|  | 1996 (7) | May | Data provided by co-author F.G-R |
| Cantiles (i, 4) | 1995 (7) | June | Data provided by co-author F.G-R |
|  | 1996 (11) | May | Data provided by co-author F.G-R |
|  | 2002 (3) | July | Porras-Peters (2004) |
| Machos (j, 4) | 1995 (20) | June | Data provided by co-author F.G-R |
|  | 2016 (7) | October | Pelayo-González et al., (2021) |
| Partido (k, 5) | 2002 (29) | July | Porras-Peters (2004) |
| Rasito (l, 5) | 1995 (9) | June | Data provided by co-author F.G-R |
|  | 1996 (15) | May | Data provided by co-author F.G-R |
|  | 2002 (21) | July | Porras-Peters (2004) |
|  | 2016 (2) | October | Pelayo-González et al., (2021) |
| San Esteban (m, 6) | 1995 (24) | June | Data provided by co-author F.G-R |
|  | 1996 (35) | May | Data provided by co-author F.G-R |
|  | 2002 (5) | July | Porras-Peters (2004) |
| San Pedro Mártir (n, 7) | 1995 (15) | June | Data provided by co-author F.G-R |
|  | 1996 (26) | May | Data provided by co-author F.G-R |
|  | 2002 (11) | July | Porras-Peters (2004) |
| San Pedro Nolasco (o, 8) | 2002 (16) | July | Porras-Peters (2004) |
| Farallón de San Ignacio (p, 9) | 2002 (23) | July | Porras-Peters (2004) |
| Los Islotes (q, 10) | 2000-2001 (164; grouped) | May to April | Cardenas-Palomo (2003) |
|  | 2002 (17) | July | Porras-Peters (2004) |
|  | 2015 (12) | July | Pelayo-González et al., (2021) |
|  | 2019 (12) | August | Pelayo-González et al., (2021) |

Details include the rookery name with corresponding letter used in Figure 1 and Zone number, data collection year, number of scat samples with identifiable taxa and season, and the corresponding publication from which the data were obtained. Frequency of Occurrence (FO) data are presented first, followed by Index of Importance (IIMP) data. For some rookeries, diet data were reported as multi-year averages over two or more years; these are labeled as “(grouped)” next to the corresponding data years.

^*^ Sources that reported only the total number of scat samples collected, without specifying how many contained hard parts with identifiable taxa.

References for citations in “Source” column are the following:

- Melin et al., 2010: Melin SR, Orr AJ, Harris JD, Laake JL, Delong RL, Gulland FMD, et al. Unprecedented mortality of California sea lion pups associated with anomalous oceanographic conditions along the central California coast in 2009. Calif Coop Ocean Fish Investig Rep. 2010;51: 182–194.
- Melin et al., 2012: Melin SR, Orr AJ, Harris JD, Laake JL, Delong RL. California sea lions: An indicator for integrated ecosystem assessment of the California current system. Calif Coop Ocean Fish Investig Rep. 2012;53: 140–152.
- Orr et al., 2011: Orr AJ, van Blaricom GR, de Long RL, Cruz-Escalona VH, Newsome SD. Intraspecific comparison of diet of California sea lions (*Zalophus californianus*) assessed using fecal and stable isotope analyses. Can J Zool. 2011;89: 109–122. doi:10.1139/Z10-101
- Lowry et al., 1991: Lowry M, Stewart B, Heath CB, Yochem P, Francis JM. Seasonal and annual variability in the diet of *Zalophus californianus* at San Nicolas Island, California, 1981-86. Fish Bull. 1991;89: 331–336.
- Lowry and Carretta 1999: Lowry M, Carretta JV. Market squid (*Loligo opalescens*) in the diet of California sea lions (*Zalophus californianus*) in southern California (1981-1995). Calif Coop Ocean Fish Investig Rep. 1999;40: 196–207.
- Lowry et al., 1990: Lowry M, Oliver C, Macky C. Food Habits of California Sea Lions *Zalophus californianus* at San Clemente Island, California, 1981-86. Fish Bull. 1990;88: 509–521.
- Cardenas-Palomo 2003: Cardenas Palomo N. Habitos alimenticios y amplitud trofica de machos y hembras adultos del lobo marino de california (*Zalophus californianus californianus*) en los Islotes, BCS, Mexico. 2003. p. tesis.
- Porras-Peters 2004: Porras-Peters H. Nivel, amplitud y superposición trófica de las colonias de lobo marino *Zalophus californianus* del Golfo de California, México. La Paz, Baja California Sur, Mexico: Centro Interdisciplinario de Ciencias Marinas; 2004.

**S3 Table. Raw paired diet and population data for California sea lions by rookery-year grouping.**

| **Rookery-year groupings** | **Zone** | **Index** | **Average diet diversity** | **Weighted energy density (kJ/gww)** | **Population change** | **Average energy density (kJ/gww)** | **Pup change (%)** |
| --- | --- | --- | --- | --- | --- | --- | --- |
| San Miguel 2000–2006 | 1 | FO | 1.70 | 5.76 | 5.03% | 5.6 | 3.2% |
| San Miguel 2009–2011 | 1 | FO | 2.00 | 4.68 | 7.80% | 5.1 | 11.6% |
| San Nicolas 1981–1986 (grouped) | 1 | FO | 1.92 | 5.66 | -2.92% | 5.36 | -4.8% |
| San Nicolas 1981–1986 | 1 | FO | 1.68 | 5.65 | -2.92% | 5.36 | -4.8% |
| San Nicolas 1981–1995 | 1 | FO | 1.95 | 5.46 | 9.47% | 5.85 | 32.0% |
| Santa Barbara 1981–1995 (grouped) | 1 | FO | 1.87 | 5.48 | 9.95% | 5.85 | 24.8% |
| San Clemente 1981–1995 (grouped) | 1 | FO | 1.81 | 5.59 | 4.51% | 5.74 | 7.7% |
| San Clemente 1981–1986 | 1 | FO | 1.96 | 5.09 | -1.61% | 5.50 | -1.9% |
| Los Islotes 1990 | 10 | FO | 2.40 | 4.59 | 2.43% | 4.7 | 5.12% |
| San Pedro Mártir 1995–96 | 7 | FO | 2.14 | 5.3 | -1.53% | 4.93 | -0.18% |
| San Esteban 1995–96 | 6 | FO | 2.04 | 5.9 | 7.29% | 5.08 | -1.52% |
| Rasito 1996 | 5 | FO | 2.48 | 5.77 | -7.60% | 5.15 | 2.31% |
| Machos 1995 | 4 | FO | 1.96 | 5.74 | -1.17% | 5.29 | 4.37% |
| Cantiles 1995–96 | 4 | FO | 1.36 | 4.51 | 6.15% | 5.05 | 6.76% |
| Granito 1995–96 | 4 | FO | 0.90 | 5.34 | 3.70% | 5.15 | 12.74% |
| Isla Lobos 1995–96 | 3 | FO | 1.93 | 5.13 | -2.03% | 5.57 | 5.16% |
| Los Islotes 2000 | 10 | FO | 2.03 | 4.69 | 7.37% | 5.00 | 3.26% |
| Rasito 2016 | 5 | FO | 2.20 | 4.31 | 7.25% | 4.31 | 13.52% |
| Machos 2016 | 4 | FO | 2.01 | 4.66 | -11.65% | 4.92 | 17.32% |
| Cantiles 2016 | 4 | FO | 2.70 | 4.42 | 7.09% | 4.58 | 7.92% |
| Granito 2016, 2018 | 4 | FO | 3.02 | 4.85 | 7.09% | 5.15 | 12.71% |
| Los Islotes 2015, 2019 | 10 | FO | 1.59 | 5.19 | -1.18% | 5.1 | 8.44% |
| San Pedro Mártir 1995–96 | 7 | IIMP | 1.526 | 5.86 | -1.5% | 5.58 | -0.2% |
| San Esteban 1995–96 | 6 | IIMP | 1.315 | 6.29 | 7.3% | 6.48 | -1.5% |
| Rasito 1995–96 | 5 | IIMP | 1.682 | 5.19 | -11.2% | 5.50 | 3.9% |
| Machos 1995 | 4 | IIMP | 1.528 | 5.94 | -1.2% | 5.75 | 4.4% |
| Cantiles 1995–96 | 4 | IIMP | 0.931 | 4.27 | 6.1% | 4.85 | 6.8% |
| Granito 1995–96 | 4 | IIMP | 0.561 | 5.30 | 3.7% | 5.37 | 12.7% |
| Isla Lobos 1995–96 | 3 | IIMP | 1.479 | 5.19 | -2.0% | 4.47 | 5.2% |
| Los Islotes 2002 | 10 | IIMP | 1.848 | 4.7 | 4.4% | 5.08 | -0.2% |
| San Esteban 2002 | 6 | IIMP | 1.503 | 5.3 | 9.2% | 5.42 | 16.3% |
| Rasito 2002 | 5 | IIMP | 0.062 | 5.43 | 5.5% | 4.85 | 12.9% |
| San Pedro Mártir 2002 | 7 | IIMP | 0.685 | 3.10 | -0.7% | 4.98 | -6.4% |
| Cantiles 2002 | 4 | IIMP | 1.096 | 3.81 | -3.6% | 3.81 | -8.8% |
| Isla Lobos 2002 | 3 | IIMP | 1.326 | 4.71 | 4.5% | 5.01 | -2.1% |
| San Pedro Nolasco 2002 | 8 | IIMP | 1.874 | 4.99 | -1.4% | 5.11 | -2.1% |
| Partido 2002 | 5 | IIMP | 1.454 | 6.81 | -7.9% | 5.29 | -3.9% |
| Rocas Consagradas 2002 | 2 | IIMP | 1.060 | 5.33 | 5.4% | 4.63 | 5.4% |
| Farallón de San Ignacio 2002 | 9 | IIMP | 2.386 | 6.20 | -3.8% | 4.94 | -5.2% |
| Los Islotes 2015 | 10 | IIMP | 0.957 | 4.38 | 1.3% | 5.03 | 8.2% |
| Rasito 2016 | 5 | IIMP | 1.921 | 4.17 | 7.2% | 4.31 | 13.5% |
| Granito 2016, 2018 | 4 | IIMP | 1.300 | 4.59 | 6.6% | 4.84 | 12.7% |
| Cantiles 2016, 2018 | 4 | IIMP | 1.433 | 5.15 | 7.1% | 4.90 | 7.9% |
| Los Islotes 2019 | 10 | IIMP | 1.684 | 6.10 | -3.7% | 5.09 | 8.6% |

For some rookeries, diet data were reported as multi-year averages over two or more years; these are labeled as “(grouped)” next to the corresponding data years.”

##### S4 Table. Raw paired diet and population data for California sea lions by Zone-era grouping.

| **Zone-era groupings** | **Era** | **Index** | **Average diet diversity** | **Population change** | **Weighted average energy density (kJ/gww)** | **Population estimate** |
| --- | --- | --- | --- | --- | --- | --- |
| Zone 1 – Channel Islands | 1981–1995 | FO | 1.87 | 2.7% | 5.49 | 9,216 |
| Zone 1 – Channel Islands | 2000–2011 | FO | 1.85 | 6.4% | 5.22 | 44,720 |
| Zone 3 – Isla Lobos | 1995–1996 | FO | 1.93 | -2.0% | 5.13 | 2,822 |
| Zone 4 – Machos, Cantiles, Granito | 1995–1996 | FO | 1.41 | 2.9% | 5.20 | 1,355 |
| Zone 4 – Machos, Cantiles, Granito | 2016, 2018 | FO | 2.58 | 0.8% | 4.64 | 696 |
| Zone 5 – Rasito | 1996 | FO | 2.48 | -7.6% | 5.77 | 362 |
| Zone 5 – Rasito | 2016 | FO | 2.20 | 7.2% | 4.31 | 308 |
| Zone 6 – San Esteban | 1995–1996 | FO | 2.04 | 7.3% | 5.90 | 7,171 |
| Zone 7 – San Pedro Mártir | 1995–1996 | FO | 2.14 | -1.5% | 5.32 | 1,963 |
| Zone 10 – Los Islotes | 1990 | FO | 2.21 | 4.9% | 4.64 | 347 |
| Zone 10 – Los Islotes | 2015, 2019 | FO | 1.59 | -1.2% | 5.19 | 538 |
| Zone 2 – Rocas Consagradas | 2002 | IIMP | 1.06 | 5% | 5.33 | 839 |
| Zone 3 – Isla Lobos | 1995–1996 | IIMP | 1.48 | -2% | 5.19 | 2,822 |
| Zone 3 – Isla Lobos | 2002 | IIMP | 1.33 | 4% | 4.71 | 1,897 |
| Zone 4 – Machos, Cantiles, Granito | 1995–1996 | IIMP | 1.01 | 3% | 5.17 | 1,355 |
| Zone 4 – Machos, Cantiles, Granito | 2002 | IIMP | 1.10 | -4% | 3.81 | 1,090 |
| Zone 4 – Machos, Cantiles, Granito | 2016, 2018 | IIMP | 1.20 | 7% | 4.87 | 729 |
| Zone 5 – Rasito, Partido | 1995–1996 | IIMP | 1.68 | -11% | 5.19 | 366 |
| Zone 5 – Rasito, Partido | 2002 | IIMP | 0.76 | -1% | 6.12 | 507 |
| Zone 5 – Rasito, Partido | 2016 | IIMP | 1.30 | 7% | 4.17 | 308 |
| Zone 6 – San Esteban | 1995–1996 | IIMP | 1.32 | 7% | 4.99 | 7,171 |
| Zone 6 – San Esteban | 2002 | IIMP | 1.45 | 9% | 6.81 | 6,334 |
| Zone 7 – San Pedro Mártir | 1995–1996 | IIMP | 1.53 | -2% | 5.86 | 7,171 |
| Zone 7 – San Pedro Mártir | 2002 | IIMP | 0.69 | -1% | 3.10 | 2,405 |
| Zone 8 – San Pedro Nolasco | 2002 | IIMP | 1.87 | -1% | 4.99 | 937 |
| Zone 9 – Farallón de San Ignacio | 2002 | IIMP | 2.39 | -4% | 6.20 | 643 |
| Zone 10 – Los Islotes | 2002 | IIMP | 1.85 | 4% | 4.72 | 404 |
| Zone 10 – Los Islotes | 2015 | IIMP | 1.92 | 1% | 4.38 | 538 |
| Zone 10 – Los Islotes | 2019 | IIMP | 1.68 | -4% | 6.10 | 659 |

S5 Table. List of 114 diet prey taxa (scientific and common name) consumed by California sea lions in the Gulf of California (MEX) or the Channel Islands (USA) showing their average energy density, category assigned, and country where data was collected.

| **Scientific name** | **Common name** | **Energy Density (kJ/gww)** | **Category** | **Country** |
| --- | --- | --- | --- | --- |
| *Abraliopsis affinis* | Squid | 4.40 | S | MEX |
| *Abraliopsis species* | Squids | 4.40 | S | USA |
| *Anisotremus davidsonii* | Xantic sargo | 4.88 | B | MEX |
| *Apogon retrosella* | Barspot cardinalfish | 4.70 | B | MEX |
| *Argentina sialis* | North-Pacific argentine | 3.57 | M | MEX |
| *Atherinops species* | Topsmelt silverside | 6.20 | SF | MEX |
| *Atherinopsis californiensis* | Jack silverside | 6.20 | M | MEX |
| *Aulopus* | Royal flagfin | 4.43 | B | MEX |
| *Aulopus bajacali* | Eastern Pacific flagfin | 4.43 | B | MEX |
| *Balistes polylepis* | Finescale triggerfish | 3.84 | B | MEX |
| *Bodianus diplotaenia* | Mexican hogfish | 3.84 | B | MEX |
| *Brosmophycis marginata* | Red brotula | 3.39 | B | MEX |
| *Calamus brachysomus* | Pacific porgy | 7.45 | B | MEX |
| *Caulolatilus princeps* | Ocean whitefish | 7.45 | B | MEX |
| *Ceratoscopelus townsendi* | Dogtooth lampfish | 7.16 | L | MEX |
| *Cetengraulis mysticetus* | Pacific anchoveta | 6.01^b^ | SF | MEX |
| *Chromis punctipinnis* | Blacksmith damselfish | 4.68 | B | USA |
| *Citharichthys species* | Flatfish | 3.33 | B | MEX |
| *Clupea pallasii* | Pacific herring | 7.51 | SF | USA |
| *Coelorinchus scaphopsis* | Shoulderspot grenadier | 5.10 | G | MEX |
| *Cololabis saira* | Pacific saury | 7.50 | M | USA |
| *Cynoscion reticulatus* | Shorefish | 7.99 | B | MEX |
| *Decapodiformes* | Superorder of squids | 4.60 | S | USA |
| *Diaphus theta* | California headlightfish | 9.88 | L | MEX |
| *Diplectrum macroposoma* | Mexican sand perch | 4.50^a^ | B | MEX |
| *Diplectrum pacificum* | Inshore sand perch | 4.03 | B | MEX |
| *Diplectrum species* | Sandperch | 5.02 | B | MEX |
| *Doryteuthis opalescens* | Opalescent inshore squid | 3.70 | S | USA |
| *Dosidicus gigas* | Humboldt squid | 5.39^a^ | S | MEX |
| *Engraulidae* | Anchovies | 6.17^a^ | SF | MEX |
| *Engraulis mordax* | Californian anchovy | 6.70 | SF | MEX |
| *Engraulis mordax* | Northern anchovy | 6.80 | SF | USA |
| *Girella nigricans* | Rudderfish | 4.40 | M | MEX |
| *Gonatopsis borealis* | Boreopacific armhook squid | 4.20 | S | USA |
| *Gonatus berryi* | Berry armhook squid | 5.02 | S | MEX |
| *Gonatus onyx* | Clawed armhook squid | 5.86 | S | USA |
| *Gonatus species* | Armhook squid | 5.90 | S | USA |
| *Haemulidae species* | Grunt fish | 4.88 | B | MEX |
| *Haemulon californiensis* | Yellowspotted grunt | 4.88 | M | MEX |
| *Haemulon flaviguttatum* | Greybar grunt | 4.88 | M | MEX |
| *Haemulon sexfasciatum* | Scaled-fin grunt | 4.88 | SF | MEX |
| *Haemulon species* | Californian salema | 4.88 | SF | MEX |
| *Haemulopsis leuciscus* | Raucous grunt | 4.88 | B | MEX |
| *Haemulopsis species* | Grunt fish | 4.88 | B | MEX |
| *Hemanthias peruanus* | Splittail bass | 4.50 | SF | MEX |
| *Hemanthias species* | Sea bass | 4.50 | B | MEX |
| *Hermosilla azurea* | Zebra perch | 4.40 | SF | MEX |
| *Holacanthus passer* | King angelfish | 7.45 | B | MEX |
| *Icelinus tenuis* | Spotfin sculpin | 5.82 | B | MEX |
| *Lepophidium prorates* | Prowspine cusk eel | 3.39 | B | MEX |
| *Lestidiops species* | Barracudina | 4.30 | SF | MEX |
| *Leuroglossus stilbius* | California smoothtongue | 3.90 | M | USA |
| *Loliolopsis diomedeae* | Dart squid | 3.75 | S | MEX |
| *Lycodes cortezianus* | Bigfin eelpout | 7.60 | B | USA |
| *Merluccius productus* | North Pacific hake | 4.20^b^ | G | MEX |
| *Merluccius productus* | Pacific Hake | 4.20^b^ | G | USA |
| *Merluccius species* | Hake | 4.07 | G | MEX |
| *Micropogonias ectenes* | Slender croaker | 7.99 | B | MEX |
| *Micropogonias species* | Croaker | 7.99 | B | MEX |
| *Myctophidae* | Lanternfish | 7.62^a^ | L | MEX |
| *Nannobrachium species* | Lanternfish | 7.63 | L | MEX |
| *Octopus rubescens* | East Pacific red octopus | 3.30 | O | USA |
| *Octopus species* | Octopus | 3.40 | O | USA, MEX |
| *Oegopsida* | Pelagic squid | 4.50^b^ | S | MEX |
| *Onychoteuthidae* | Hooked squid family | 5.40 | S | USA |
| *Onychoteuthis borealijaponicus* | Boreal clubhook squid | 5.48 | S | USA |
| *Ophidion scrippsae* | Basketweave cusk-eel | 3.39 | B | MEX |
| *Ophidion species* | Cusk-eels | 3.39 | B | MEX |
| *Ophistonema species* | Herrings | 7.47 | SF | MEX |
| *Orthopristis reddingi* | Bronze-striped grunt | 4.88 | SF | MEX |
| *Oxylebius pictus* | Painted greenling | 4.44 | B | MEX |
| *Paralabrax clathratus* | Kelp bass | 4.45 | M | MEX |
| *Paralabrax species* | Rock bass | 4.45 | B | MEX |
| *Paralichthys californicus* | California halibut | 3.81 | B | MEX |
| *Physiculus nematopus* | Charcoal mora | 4.00 | G | MEX |
| *Physiculus species* | Codling | 4.00 | G | MEX |
| *Pleuroncodes planipes* | Pelagic red crab (lobster) | 6.70 | C | USA |
| *Pontinus furcirhinus* | Red scorpionfish | 3.19 | B | MEX |
| *Pontinus species* | Scorpionfish | 3.19 | B | MEX |
| *Porichthys notatus* | Plainfin midshipman | 3.36 | B | MEX |
| *Porichthys species* | Midshipman | 3.36 | B | MEX |
| *Prionotus species* | Searobin | 4.63 | B | MEX |
| *Prionotus stephanophrys* | Lumptail searobin | 4.63 | B | MEX |
| *Pronotogrammus eos* | Bigeye bass | 4.45 | B | MEX |
| *Pronotogrammus multifasciatus* | Threadfin bass | 4.45 | B | MEX |
| *Sarda lineolata* | Pacific bonito | 7.04 | SF | MEX |
| *Sardinops caeruleus* | California pilchard | 7.47^a^ | SF | MEX |
| *Sardinops sagax* | South American pilchard | 7.50 | SF | MEX |
| *Sardinops sagax* | Pacific sardine | 7.50 | SF | USA |
| *Scomber japonicus* | Chub mackerel | 6.80 | SF | MEX |
| *Scomber japonicus* | Pacific mackerel | 6.80 | SF | USA |
| *Scopelengys tristis* | Pacific blackchin | 7.62 | M | MEX |
| *Scorpaenidae* | Scorpionfish | 3.19 | M | MEX |
| *Sebastes exsul* | Buccaneer rockfish | 5.51^a^ | R | MEX |
| *Sebastes jordani/species* | Rockfish | 5.60 | R | USA |
| *Sebastes macdonaldi* | Mexican rockfish | 5.51^a^ | R | MEX |
| *Sebastes species* | Rockfish | 5.60 | R | MEX |
| *Selar crumenophthalamus* | Bigeye scad | 6.27 | SF | MEX |
| *Serranus aquidens/aequidens* | Deepwater serrano | 4.45 | B | MEX |
| *Specieshyraena argentea* | Pacific barracuda | 3.36 | M | MEX |
| *Stenobrachius leucopsarus* | Northern lampfish | 9.70 | L | USA |
| *Strongylura exilis* | Californian needlefish | 6.20 | SF | MEX |
| *Symbolophorus californiensis* | Bigfin lanternfish | 7.07 | L | MEX |
| *Symphurus fasciolaris* | Banded tongue fish | 4.00 | B | MEX |
| *Symphurus species* | Tongue fish | 4.00 | B | MEX |
| *Synodus lucioceps* | California lizardfish | 4.43 | B | MEX |
| *Synodus species* | Lizardfish | 4.25 | B | MEX |
| *Trachurus species* | Jack mackerel | 6.30 | SF | MEX |
| *Trachurus symmetricus* | Pacific jack mackerel | 6.27 | SF | MEX |
| *Trachurus symmetricus* | Jack mackerel | 6.30 | SF | USA |
| *Trichiurus lepturus* | Largehead hairtail | 4.76 | M | MEX |
| *Trichiurus nitens* | Pacific cutlassfish | 5.05 | M | MEX |
| *Triphoturus mexicanus* | Mexican lampfish | 7.07 | L | MEX |
| *Zaniolepis species* | Combfish | 7.60 | B | USA |

Some species had the same scientific name, but different common names depending on the region. Categories assigned based on broad ecological characteristics as per Trites et al., 2007 [40]: B: benthic species, C: crustaceans, G: gadids, L: lanternfish, O: octopus, R: rockfish, SF: schooling fish, S: squid, M: miscellaneous.

^a^ Species for which energy density values were not available at the species level and the value used was an average from the same family.

^b^ Species for which energy density values were not available at the species level and the value used was an average from a closely related family.
